# Special Report: The Biology of Inequalities in Health: The Lifepath Consortium

**DOI:** 10.3389/fpubh.2020.00118

**Published:** 2020-05-12

**Authors:** Paolo Vineis, Mauricio Avendano-Pabon, Henrique Barros, Mel Bartley, Cristian Carmeli, Luca Carra, Marc Chadeau-Hyam, Giuseppe Costa, Cyrille Delpierre, Angelo D'Errico, Silvia Fraga, Graham Giles, Marcel Goldberg, Michelle Kelly-Irving, Mika Kivimaki, Benoit Lepage, Thierry Lang, Richard Layte, Frances MacGuire, Johan P. Mackenbach, Michael Marmot, Cathal McCrory, Roger L. Milne, Peter Muennig, Wilma Nusselder, Dusan Petrovic, Silvia Polidoro, Fulvio Ricceri, Oliver Robinson, Silvia Stringhini, Marie Zins

**Affiliations:** ^1^Department of Epidemiology and Biostatistics, Imperial College London, London, United Kingdom; ^2^Department of Social Sciences, Health and Medicine, King's College London, London, United Kingdom; ^3^EPIUnit – Institute of Public Health University of Porto, Porto, Portugal; ^4^Department of Epidemiology & Public Health, University College London, London, United Kingdom; ^5^Center for Primary Care and Public Health (UNISANTE), University of Lausanne, Lausanne, Switzerland; ^6^Zadig, Milan, Italy; ^7^Department of Clinical Science & Biology, Turin University Medical School, Turin, Italy; ^8^UMR LEASP, Université de Toulouse III, UPS, Inserm, Toulouse, France; ^9^Department of Epidemiology, ASL TO3, Turin, Italy; ^10^Cancer Epidemiology Division, Cancer Council Victoria, Melbourne, VIC, Australia; ^11^Centre for Epidemiology and Biostatistics, Melbourne School of Population and Global Health, The University of Melbourne, Melbourne, VIC, Australia; ^12^Precision Medicine, School of Clinical Sciences at Monash Health, Monash University, Melbourne, VIC, Australia; ^13^UMS 011 Inserm - UVSQ ≪ Cohortes épidémiologiques en population ≫, Villejuif, France; ^14^Department of Epidemiology and Public Health, University College London, London, United Kingdom; ^15^Department of Sociology, School of Social Sciences and Philosophy, Trinity College Dublin, Dublin, Ireland; ^16^Department of Public Health, Erasmus MC, University Medical Center Rotterdam, Rotterdam, Netherlands; ^17^Department of Medical Gerontology, Trinity College Dublin, Dublin, Ireland; ^18^Mailman School of Public Health, Columbia University, New York, NY, United States; ^19^Molecular Epidemiology and Exposomics Unit, Italian Institute for Genomic Medicine, Turin, Italy; ^20^Unit of Population Epidemiology, Division of Primary Care, Geneva University Hospitals, Geneva, Switzerland

**Keywords:** social inequalities, socioeconomic position, healthy aging, life-course, omics, biology

## Abstract

Funded by the European Commission Horizon 2020 programme, the *Lifepath* research consortium aimed to investigate the effects of socioeconomic inequalities on the biology of healthy aging. The main research questions included the impact of inequalities on health, the role of behavioral and other risk factors, the underlying biological mechanisms, the efficacy of selected policies, and the general implications of our findings for theories and policies. The project adopted a life-course and comparative approach, considering lifetime effects from childhood and adulthood, and pooled data on up to 1.7 million participants of longitudinal cohort studies from Europe, USA, and Australia. These data showed that socioeconomic circumstances predicted mortality and functional decline as strongly as established risk factors currently targeted by global prevention programmes. Analyses also looked at socioeconomically patterned biological markers, allostatic load, and DNA methylation using richly phenotyped cohorts, unraveling their association with aging processes across the life-course. *Lifepath* studies suggest that socioeconomic circumstances are embedded in our biology from the outset—i.e., disadvantage influences biological systems from molecules to organs. Our findings have important implications for policy, suggesting that (a) intervening on unfavorable socioeconomic conditions is complementary and as important as targeting well-known risk factors, such as tobacco and alcohol consumption, low fruit and vegetable intake, obesity and a sedentary lifestyle, and that (b) effects of preventive interventions in early life integrate interventions in adulthood. The report has an executive summary that refers to the different sections of the main paper.

## Executive Summary

### Introduction

More people are living longer, and living longer in retirement, but they are not necessarily spending their later years in good health. It is well-established that individuals from disadvantaged income, education, or occupational grade develop diseases earlier, experience more years with disability, and die younger than their more advantaged peers (1, 2). People with socioeconomic disadvantage are more likely to suffer from worse health in the life-course.

The risk of poor health tends to increase with reductions in socioeconomic position (SEP), creating what is known as a social gradient in health. In principle, if the richer can achieve healthy aging it should be possible to achieve healthy aging for all. Affluence, however, is more than financial capital. It includes access to high-quality education, meaningful employment, good housing, transport (both public and private), high-quality greenspace, and many other features.

*Lifepath* is a research consortium funded by the European Commission under Horizon 2020, designed to explore whether healthy aging is an achievable goal for the entire society. If we can develop a highly nuanced understanding of how social conditions alter human biology to produce disease, then we may also better understand how to address poverty-associated disease. The main goals of *Lifepath* are shown in the Box1 below. The *Lifepath* project has produced a series of studies which have integrated European-wide data on SEP, environmental exposures and behavioral risk factors with health and other biological measurements (3). *Lifepath* has examined how SEP influences health, partly through behaviors such as smoking, unhealthy diets, sedentary lifestyles, and physical or chemical exposures from pollution and occupational exposures. In addition, the circumstances in which children are born and grow up strongly influence health. *Lifepath* researchers used a number of different indicators such as educational attainment, occupation, place of residence, and level of income to measure SEP. Harmonized data on occupational class, education, father's occupational class and income were gathered from 11 adult cohorts and seven child cohorts (4) (*see the section “What is socio-economic position and how it is measured,” page 9, for theoretical foundations; and the section “Life-course model of healthy aging,” page 11, for the concept of life-course epidemiology*).

Box 1 ***Lifepath* goals.**

•The main research questions in *Lifepath* included the impact of inequalities on health, the role of behavioral and other risk factors, the underlying biological mechanisms, the efficacy of selected policies, and the general implications of our findings for theories and policies. We first set the theoretical foundations on page 9.
•An overarching goal was to show that healthy aging is an achievable goal for society as a whole, as it is already experienced by individuals of high socioeconomic position (SEP)(see in particular Lifepath Results on page 14).
•We aimed to improve the understanding of the mechanisms through which healthy aging pathways diverge by SEP, by investigating life-course biological pathways using *omic* technologies (section starting on page 18).
•Another goal was to examine the consequences of economic recession on the health of children and families, and the impact of social policies on health inequalities (section starting on page 25).
•The overarching aim was to produce up-to-date, relevant and original evidence for healthy aging policies, in particularly “health in all policies” (page 27–34).

Population studies across Europe confirmed the existence of socioeconomic differences in health. Inequalities in mortality and morbidity among socioeconomic groups are a highly persistent phenomenon despite having been the focus of public health policy in many countries (*see the section “Health inequalities in European populations and their determinants,” page 18*). Population data also pointed at variation between countries and genders in the size and time trends of socioeconomic differences. The inequalities in mortality and disability-free life expectancy were larger in Central & Eastern Europe than in other regions of Europe and more so among men. Relative inequalities in mortality (measured e.g., as rate ratios between a lower and higher educational group) have almost universally increased over the past decades, while absolute inequalities in mortality have often declined or remained stable. This also holds for specific causes, such as cardiovascular disease, which are amenable to policy and health care. *Lifepath* found that the top three risk factors that contributed most to educational differences in life expectancy and disability-free life expectancy are smoking, low income and high body weight.

*Lifepath* investigated biological pathways underlying social differences in healthy aging. The project integrated social science approaches with biology (including molecular epidemiology) using existing population cohorts and omics measurements (particularly epigenomics). The ultimate goal of *Lifepath* was to generate scientific evidence that could inform policy measures to reduce the impact of socioeconomic disadvantage on health. Better understanding of the biological basis of how social determinants influence health could be used to develop future evidence-based health policies and strategies.

Finally, *Lifepath* exploited the natural experiment generated by the economic recession to understand how economic shocks influenced the health of children and families and how major changes in social policies that shaped educational opportunities and reduced poverty affected the health of entire cohorts of Europeans. *Lifepath* integrated longitudinal observational data with results of trials of social programmes in order to understand how health inequalities can be reversed by intervening on social determinants.

### Socioeconomic Position as a Risk Factor

According to the World Health Organization (WHO), most premature deaths due to non-communicable diseases (NCDs)—including cardiovascular diseases, cancers, chronic respiratory diseases, and diabetes—are associated with common risk factors, namely smoking, high alcohol consumption, poor diet, physical inactivity, raised blood pressure, and high salt consumption (5). In addition, WHO considers air pollution to be a modifiable risk factor of significant concern (https://www.who.int/airpollution/en/).

It is well-recognized that SEP may influence health through behaviors such as smoking, poor diet, sedentary lifestyle, or exposure to pollution in occupational or household environments. However, social circumstances are important determinants of mortality and accelerated aging in their own right (6). Despite this evidence, key international health strategies—including the 2013-20 WHO Global Plan for the Prevention and Control of Non-Communicable Diseases (NCDs) and the Global Burden of Disease (GBD) Collaboration—do not include socioeconomic disadvantage as a modifiable risk factor.

**Figure 1:**
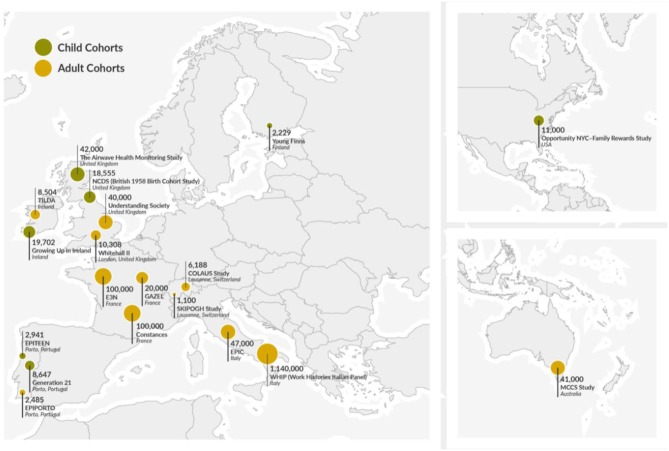
Geographic location of the cohorts used in Lifepath.

NCDs are characterized by several common attributes, such as chronicity, global burden and a preventable nature (3). They share common risk factors, such as high cholesterol, blood pressure and glucose, and risky behaviors. The burden of NCDs is explained by economic, social, environmental conditions, injustice and the distribution of inequality. *Lifepath* researchers explored SEP as a risk factor for adult NCDs in a multi-cohort study of over 1.7 million individuals from 48 independent cohorts from the UK, France, Switzerland, Portugal, Italy, the USA, and Australia (7) (Figure 1) (*see the section “Health inequalities in cohorts and the underlying mechanisms,” page 18*). SEP was measured as occupational status in three categories: high (higher professionals and managers, higher clerical, services and sales workers), intermediate (small employers and self-employed, farmers, lower supervisors and technicians) or low (lower clerical, services and sales workers, skilled workers and semi-skilled and unskilled workers) and was compared to six risk factors: tobacco use, alcohol consumption, insufficient physical activity, raised blood pressure, obesity and diabetes. This investigation is apparently the first large-scale study to directly compare the importance of socioeconomic circumstances as determinants of health with six of the major risk factors targeted in global health strategies for the reduction of premature mortality. Low SEP was associated with 2.1 years of life lost (YLL) between ages 40 and 85 years and was in the same range as YLL from the other six risk factors (Table 1).

**Table 1:** Years of Life Lost associated with 6 major risk factors and disadvantaged SEP (7).

**Mediating factor**	**Years of life lost (YLL)**
Alcohol (high use)	0.5
Diabetes	3.9
Hypertension	1.6
Obesity	0.7
Physical inactivity	2.4
Smoking	4.8
SEP	2.1

### Healthy Aging Over the Life-Course

Human populations are aging in most countries. Consequently, policy makers around the world are increasingly considering healthy aging to be a public health priority so as to reduce the burden of ill-health in old age on populations, healthcare services, and national economies. In addition to preventing premature mortality, there is a growing need to reduce time spent living in poor health (8). We explored the association between socioeconomic position and walking speed—used as a measure of physical functioning—in old age in cohorts from Europe, the United States, Latin America, Africa, and Asia (*see the section “Health inequalities in cohorts and the underlying mechanisms,” page 18*). Years of functioning lost (YFL) due to disadvantaged SEP were comparable to YFL due to the well-recognized risk factors of obesity, diabetes, low physical activity, and were greater than YFL due to high alcohol intake, tobacco smoking, and hypertension. YFL associated with disadvantaged SEP were higher in the United States than in Europe. Overall, disadvantaged SEP was associated with the loss of 6 years of physical functioning.

**Figure 2:**
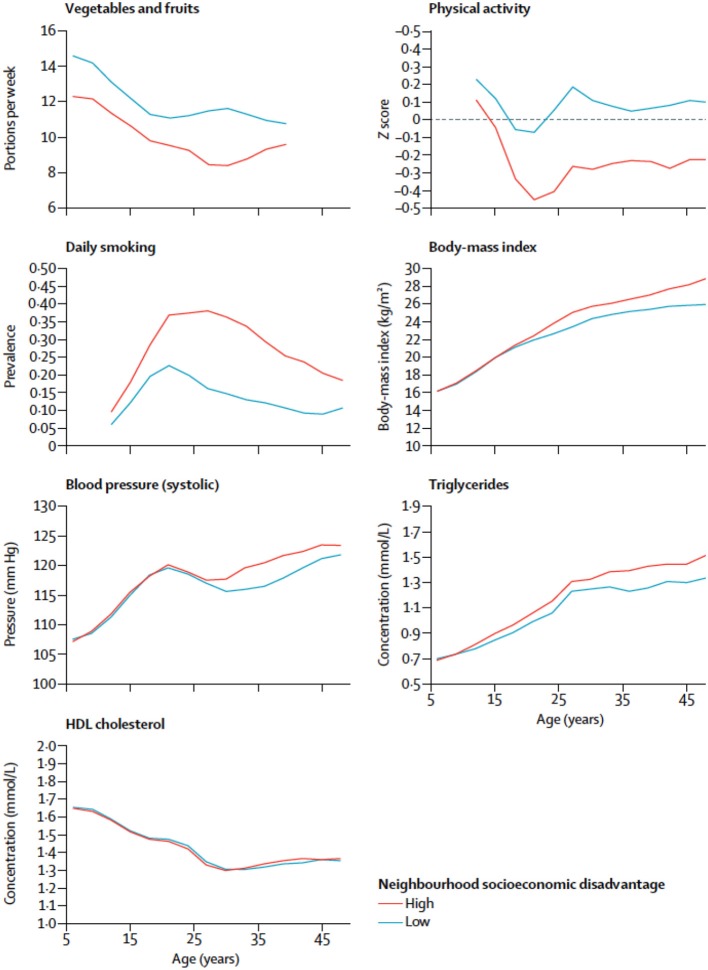
Risk factors for cardiometabolic disease by age and cumulative neighborhood socioeconomic disadvantage. The x-axis is age (years) and the y-axis is the prevalence of risk factors for cardiometabolic diseases at different ages from Kivimaki et al. (10).

In a cohort study of cardiometabolic multimorbidity, midlife socioeconomic factors were found to be important predictors of two or more cardiometabolic conditions such as diabetes, stroke, or coronary heart disease in participants aged 50, even taking into account clinical and behavioral risk factors (9) (Figure 2). This is an important finding as it contributes to an understanding of how disease progresses with age toward multimorbidity, which is associated with poor quality of life, higher healthcare costs, and increased mortality. Such understanding is highly relevant to prevention efforts and policies to reduce socioeconomic disadvantage in middle and older age.

Disadvantaged SEP has also been associated with depression, particularly in women and younger adults, and with dementia, as shown by results from, respectively, the Constances cohort (11) and the Whitehall II cohort (12). The relationships between mental and cognitive health and work are complex. For example, it has been shown that working beyond retirement age (13) was associated with occupational class, though continuing working can also be related to the quality of the jobs, i.e., cognitive health and work/SEP may have a two-way relationship.

### Early Life and Childhood

*Lifepath* research indicates that socioeconomic circumstances cast a long shadow from the womb to the tomb. Disadvantaged SEP at birth, in early years and childhood negatively influences health in adulthood and old age. We investigated the relationship between socioeconomic position (measured by maternal education) and obesity by analyzing body mass index (BMI) from over 41,399 children in four prospective cohort studies: Generation XX1 (G21—Portugal), Growing Up in Ireland (GUI—Ireland) infant and child cohorts, and the Millennium Cohort Study (MCS—UK) (14). We found that childhood overweight and obesity was present in all social groups and there was a higher prevalence of overweight and obesity among girls compared to boys. However, the burden of overweight and obesity was concentrated in children from lower socioeconomic backgrounds. Socioeconomic differentials in overweight and obesity emerged in early childhood (as early as at age three) and widened over time. Such a result is similar to previous estimates indicating that the social gradient in BMI emerges as early as 9 months of age (15), or 3 or 4 years depending on the studies (16, 17). This provides important evidence about the timing of potential policy interventions designed to reduce emerging obesity in childhood. For example, research indicates that around half of the social class differential in obesity risk in early life reflects patterns of breastfeeding and early weaning onto solid foods (16). Action is needed because being obese or overweight is associated with an increased risk of type 2 diabetes, hypertension, and cardiovascular disease (18). Furthermore, the importance of such action is highlighted by other researchers who illustrate that an association between being overweight and higher incidence of cancer begins in early life (19, 20).

The burden of obesity also has implications for the delivery and cost of healthcare across the life-course. In a recent meta-analysis of 200,000 children, it was predicted that 55% of obese children will become obese adolescents and 80% of obese adolescents will become obese adults (21). Other researchers have investigated the degree to which SEP impacts on weight gain and have found a number of risk factors that were consistently associated with childhood obesity in early life (22). These include high maternal BMI, prenatal exposure to tobacco smoke, excessive maternal weight gain during pregnancy, bottle feeding (23), and early transition to solid foods (24). All these are influenced by SEP.

In addition, children from more disadvantaged backgrounds were more likely to be infected by Epstein Barr virus due to their living conditions (25), and they have a stronger likelihood of suffering from later-life chronic kidney disease (26).

**Figure 3:**
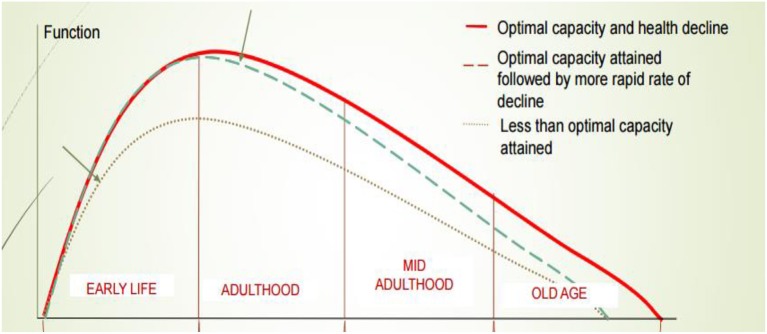
Health trajectories in the life-course. The x-axis is age and the y-axis is a theoretical measure of function in different organs.

Disadvantaged socio-economic position in early life (even before birth) may condition lifestyles and health-related behaviors, subsequently affecting health in adulthood (Figures 2, 3). Lower SEP in early life may be associated with suboptimal early life nutrition, tobacco exposure *in utero* and infancy, slower fetal growth or premature birth. The child's development is clearly sensitive to the surrounding environment in early childhood and to the availability of economic resources. Psychosocial stress is thought to affect brain development in childhood by affecting glucocorticoid and catecholamine levels which affect executive function, emotional and behavioral control and analytical thinking. Low socioeconomic advantage in childhood may result in educational disadvantage, which in turn drives economic disadvantage in adulthood. As a whole, this evidence has important implications for health and social policy.

### Effect of Recession and Austerity Measures on Inequalities in Health

According to a large amount of research—including evidence from *Lifepath*—there is a strong relationship between SEP and health, but establishing the causal relationship between these two elements can often be difficult. One of *Lifepath's* central objectives was to evaluate the impact of the 2008 recession in Europe as a way of assessing the causal relationship between exposure to low levels of economic resources and health, since many households who had been relatively stable before the crisis, later found themselves experiencing job loss, low income, and economic strain. Periods of economic recession therefore provide an opportunity to explore the impact of a change of SEP on health.

*Lifepath* researchers (*see the section “Economic downturn: the evidence from Lifepath,” page 25*) examined the impact of the 2008 recession among Irish children within the Growing Up in Ireland Infant Cohort Study before (2008), during (2011), and after the recession (2013), incorporating questions on the effects of the financial crisis on families (27). A decrease in welfare benefits during the recession was associated with a significant increase in the risk of asthma and atopy (a tendency to develop hyper-allergenic conditions such as hay fever and eczema). While the loss of parental jobs was not associated with child health, a decrease in working hours was associated with increased reports of “fair” or “poor” child health. These results suggest that vulnerable families and children should receive additional support during times of recession and that failing to protect such families may have long-term implications for child health. Ways to provide income support for families experiencing economic hardship during recession should be developed. *Lifepath* research also suggested a possible mechanism through which experience of recession influenced atopic disease: both parents and children in households that experienced more economic strain were more likely to show higher levels of psychosocial stress and an increased risk of depression (28).

Mackenbach et al. (29) completed a study of trends in health inequalities in 27 European countries, which included the period of the 2008 banking crisis. The recession caused a rise in unemployment levels and fiscal austerity in most European countries. Unemployment rates were particularly high in Ireland, Spain, Portugal, Greece, and Cyprus. Previous recessions have seen a rise in unemployment and short-term increases in deaths from suicide and alcohol-related health problems. In the 2008 recession, countries that were particularly badly hit such as Greece, Spain, and Portugal saw an increase in suicide rates and poor mental health (30).

The study by Mackenbach et al. (29) is thought to be the largest study of trends in health inequalities conducted to date. Data were analyzed on total and cause-specific mortality from 1980 to 2014 for 17 countries and survey data on self-assessed health and activity limitations from 2002 to 2014 for 27 countries. In most Western European countries, mortality continued to decline across the SEP gradient, measured by education. However, mortality from causes related to smoking went up for younger less educated women and mortality from causes related to alcohol increased among less educated men and women. No Western European country experienced an increase in mortality comparable to the increased rates seen in the US during the global recession. In Eastern European countries, such as Hungary, Lithuania, and Estonia, mortality started to decline among those with less education. Levels of mortality still remain high, however, particularly among the middle-aged.

The study indicated no short-term impact of the economic crisis on health inequalities at the population level, though it may have been statistically under-powered for detecting the full health effects of the economic crisis. Most European countries have experienced a mortality decline for several decades and the evidence suggests this was not affected by the recession. This likely reflects a level of stability in most European countries based on the provision of financially accessible health care and social support systems. However, as showed in the case of Ireland, nations should tackle the negative effects of deprivation during recessions.

### Biological Mechanisms—Allostatic Load and Biological Markers

*Lifepath* project also explored how disadvantaged SEP can be associated with poor health by looking at the intermediate biological processes. Social disadvantage in early life may cause chronic biological changes such as increased inflammation (31), which can lead to a range of health conditions such as cardiovascular disorders, asthma, and cancer (32).

*Studies* on multiple biomarkers and omics provided credible mechanisms for our conceptual life-course model, including epigenetics, inflammatory markers, allostatic load, and metabolomics (*see the section “Health inequalities in cohorts and the underlying mechanisms,” page 18*). Some (early) changes seem to be responsive/adaptive, but in the long run they become pathological. Resilience (i.e., adaptation to environmental challenges) has a cost, meaning that it creates a debt that may be paid later in the life-course in terms of disease.

*Lifepath* researchers found that disadvantaged SEP can create long-lasting psychosocial stress with chronic effects through physiological wear-and-tear involving inflammatory responses, impaired immune function and epigenetic acceleration of aging (14). Differences in SEP are revealed in cells, tissues and organs causing earlier disease onset and mortality among more socially disadvantaged groups. One approach taken by *Lifepath* is to examine allostatic load, a composite (multi-variable) measure of overall physiological strain (wear-and-tear). Data from the 1958 British birth cohort indicated that lower maternal education and manual paternal occupation were associated with a higher allostatic load at 44 years (33). The research suggests a pathway where parental occupation and education affect children's educational attainment and this impacts on later life. Psychosocial adversity during childhood, described as adverse childhood experiences (ACE), was also related to allostatic load at 44 years (6, 34) in the 1958 British birth cohort. Path analysis models showed that the association of ACE with allostatic load was strongly mediated by health behaviors (principally smoking) at 23 years and socioeconomic position (through education level at 23 years and SEP at 33 years).

Allostatic load is a composite measure influenced probably by stress, diet, infection, risk behaviors, and other variables, thus it is currently hard to determine what the implications for policy are. A more specific inflammatory marker, connected to the allostatic load score, is the C-reactive protein (CRP). A study conducted among 18,349 individuals from Britain, Ireland, Portugal, and Switzerland (35) showed that SEP could affect health via the concentration of CRP and that mean inflammation levels were highest in Portugal, the country with the highest income disparities and lowest in Switzerland, the lower income inequality country. Another study by Berger et al. (36) has shown that socioeconomic disadvantage in young adults is associated with later life CRP concentrations. The research was conducted in more than 23,000 individuals from 3 countries (Britain, Italy, and Switzerland), and revealed a significant association between educational attainment and inflammation levels in adulthood, with BMI standing out as an important intermediate factor between SEP and inflammation.

Other *Lifepath* analyses focus specifically on how differences in SEP are revealed in the DNA of our cells. DNA methylation is used to represent overall biological aging and has been linked with educational attainment of individuals (37, 38). Disadvantaged SEP was associated with accelerated aging. The results suggest that biological aging has been faster in individuals with fewer years of education. Individuals who experienced life-course SEP improvement had intermediate levels of accelerated aging compared to extreme SEP categories, suggesting a possible reversibility of the effect that could be of great importance in terms of policy making. The relevance of this measure is supported by a study by Dugué et al. (39), which showed that the age acceleration DNA-based indicator was able to predict cancer mortality longitudinally in the course of the follow-up. This study provides increasing evidence for the existence of social-to-biological processes that go beyond the behavioral element. Socioeconomic adversity is associated with accelerated epigenetic aging, which involves biomolecular mechanisms that may link SEP to age-related diseases and longevity. *Lifepath* researchers (40) used data from the Avon Longitudinal Study of Parents and Children (ALSPAC) birth cohort to investigate the DNA methylation changes in response to early life SEP experiences (maternal and paternal education and occupation) at three time stages: birth, childhood, and adolescence. Maternal education appeared to significantly affect methylation at birth and it was suggested that the association between maternal SEP and offspring methylation is driven by mechanisms during pregnancy.

Together, these findings show the value of using biological markers to understand the relationship between social factors and health. Preclinical stages of diseases can be identified by using markers such as DNA methylation and composite indicators like the allostatic load. Biomarkers can be used to explore the impacts of income inequality. More unequal societies are thought to produce higher levels of stress in response to “status anxiety” at the individual level. A growing amount of evidence highlights the role of chronic inflammation in this connection, as described below.

### Policy Interventions to Reduce Inequalities

Appropriate policies can prevent and even reverse social-to-biological transitions, resulting in healthier aging. The conclusions of *Lifepath* analyses aim to contribute to fill knowledge gaps, thus influencing policy, notably about the biological consequences of social and economic circumstances. Some examples are reported in the main text below (*see in particular the sections “Health Impact Assessment through microsimulation models, page 27,” and “Policies to address health inequalities and the example of ACEs,” page 29*). With the exception of conditional cash transfers and compulsory schooling laws (*see section “Lessons from RCTs and non-experimental intervention studies,” page 32*), *Lifepath* has not compared or assessed different policy options or interventions. The scientific evidence that has emerged from *Lifepath* is a first step in understanding aging throughout the life-course. The policy response is complex as it needs to fit with existing policy strategies in different countries. One size will not fit all. Governments have a range of policy instruments including income support, anti-smoking legislation (such as smoke free public places), taxes (e.g., sugar tax, alcohol tax), regulation (pollution controls for emissions), and nudging (e.g., displaying healthy food options near shop check-out points).

### Conditional Cash Transfers

Conditional cash transfers (CCT) seek to reduce short-term poverty and to break intergenerational poverty by providing people on low income with a cash sum in exchange for the pursuit of positive health behaviors. These could include primary health care visits for children to ensure vaccination and growth monitoring. Cash transfer programs pursue the twin objectives of reducing immediate financial hardship while promoting parental investment in their own and their children's health and well-being. However, CCT programmes are very specific in their impact, only providing benefits associated with each programme's conditionalities (41). There may not be a “spill-over” effect to broader determinants of children's health, and programmes should be better designed to motivate parents and families to invest in the wider determinants that affect children's health (41, 42). In addition, the impact of such programs may be short-lived.

Using data from a major randomized experiment in the US, *Lifepath* is one of the first studies to show that it is possible to use randomized controlled trials of social policy to examine how changes in SEP, particularly income, influence the health of adults and children in the short- to medium-term. Using data from the NYC Family Rewards experiment, which tested a programme that provided poor families in NYC with cash transfers on the condition that they engaged in various behaviors, we were able to show improvements in psychological well-being, as well as small but significant changes in self-rated health in response to this programme. Nevertheless, changes in self-rated health appeared to be short-lived and were not sustained a few years after the programme ended. On the contrary, improvements in psychological well-being seem to take time and were observed only 42 months after the end of the study. Overall, the findings from this report offer a mixed picture of the potential of CCTs to reduce health inequalities. On the one hand, the findings suggest that conditional cash transfers may improve the psychological well-being of low-income adults, but they also suggest that effects on physical and overall health assessments of adults and children are weak or inconsistent in the short- to medium-term.

### A Cautionary Tale on the Impact of Education on Healthy Aging: Quasi-Experimental Evidence From Compulsory Schooling Laws

Findings from quasi-experimental studies in *Lifepath* (43) suggest that social policies may sometimes be effective by changing the distribution of education, in terms of cognitive aging, physical health, and functioning outcomes (44). Yet, our analyses in the French Constances cohort also offer a cautionary tale of the dangers of changing the distribution of socioeconomic position without considering potential negative mental health effects on those affected. Our analyses showed that although compulsory schooling laws increased the length of schooling and in some cases educational attainment, they may also have led to unexpected increases in depressive symptoms, and some negative effects on biological markers of diseases. These results raise questions about simple causal interpretations of the relationship between education and health. Overall, our findings suggest that changes in schooling may not always lead to expected improvements in population health, and they emphasize the need to monitor how specific social policies influence health and aging trajectories of individuals and families. However, these studies were conducted in a French cohort and the results may reflect the specific context and a specific time period.

### An Integrated Approach

In a nutshell, trajectories toward poor health can be modified by changing both intermediate unhealthy behaviors and social deprivation as such (including its impact on stress), from the very beginning of life. The two types of trajectories seem to be complementary. For policy purposes, the points above suggest (a) that the effects of prevention interventions in early life may be complementary and quantitatively comparable to interventions in adulthood, as suggested by our microsimulation models; (b) that intervening on poor socio-economic conditions is complementary and quantitatively comparable to modifying risk factors. Examples are reported in the main text below (*see the section “Health Impact Assessment through microsimulation models,” page 27*).

### The Right Interventions at the Right Time and on the Right Variables

*Lifepath* has showed that interventions to reduce health disparities are needed both in childhood, to support healthy aging through the life-course, and later in life, to help people in middle or old age who are aging in a deprived setting or need help to address their disadvantage in functioning. Each stage of life needs specific interventions that should take into account the fact that lives are linked across generations, and other aspects such as context, timing, agency and opportunity.

**Early Life** Poor health trajectories related to disadvantaged SEP start in early life, i.e., the biological effects of early exposure begin well before a person has fully taken up health behaviors like smoking. Nonetheless, by sharing a household with adults who already have unhealthy behaviors, children are exposed to these risk factors anyway. *Lifepath* outcomes all raise the major problem of the obesogenic and pro-inflammatory environments in early life and the need for prevention. Evidence also suggests that expenditure and investment in early years could be more effective and cheaper than interventions (45) or amelioration later in life.

**Early adulthood** Young adults with disadvantaged social characteristics already show a higher biological risk when compared to their more advantaged counterparts (46) and this is likely to track forwards. This biological risk is accelerated by unhealthy behaviors and living in deprived neighborhoods is associated with differences in risks for health across the life-course, including hazardous lifestyle factors from childhood and adolescence onwards, and worse glucose metabolism from early adulthood. The impact of early life social disadvantage on biology may amplify from early adulthood by age 25 (10). Tackling social exposures and health behaviors early in adulthood can limit their long-term effects and mitigate exacerbations.

**Mid adulthood** Based on *Lifepath* studies, we know that by mid-adulthood premature mortality disproportionately affects socially disadvantaged people (7), and that social patterning in physical functioning (8), physiological wear-and-tear (13, 32, 36), and in molecular processes including epigenetic age acceleration (37, 38) is observed. All these are also mediated by BMI, smoking and metabolic disorders, such as fatty liver disease and diabetes (10).

At this age we are interested in harm reduction mitigating the impacts of previous exposures upon the adults affected in terms of social exposures and behaviors. These same adults are likely to be parents and carers, therefore are part of the “exposome” of other people, notably children and adolescents. Moreover, reducing psychosocial stress as a consequence of disadvantaged SEP is as important as improving living conditions. Social welfare systems should seek stability in incomes and avoid “cliffs” where incomes change dramatically through events (decommodification). Communities need services and support, not just passive welfare.

### Key Policy Messages

The *Lifepath* project suggests that socioeconomic circumstances should be included among the risk factors targeted by global health strategies, as their impact on premature mortality and functional health is quantitatively similar—in strength and consistency across countries—to the impact of behavioral risk factors, such as smoking, obesity, and physical inactivity. This adds weight to the argument that in order to improve life expectancy and later life functioning it is necessary to reduce social disparities.

In addition, the *Lifepath* project provides evidence that poor health trajectories related to disadvantaged SEP start in early life. Studies also indicate that psychosocial stress following adverse experiences, in particular among children and vulnerable adult groups, is likely to be an important factor. Intervening at an early age seems to have an impact on poor health in middle age quantitatively similar to intervening on smoking, according to our health impact assessment models described below in the main text.

The impact of socioeconomic condition on premature aging is mediated by known behavioral and clinical factors and intermediate molecular pathways that *Lifepath* studies have revealed, including epigenetic clocks (age acceleration), inflammation, allostatic load, and metabolic pathways—highlighting the biological imprint (embodiment) of social variables and strengthening causal attribution.

Research on the impact of recessions suggests that the economic strain imposed by short-term fluctuations in resources is harmful over the long-term. Social protection systems should be designed to reduce the volatility of household incomes by offering short-term income protection, and, potentially, investment in labor and human capital to ensure long-term income maintenance.

The *Lifepath* research revealed a number of associations between poor maternal and paternal SEPs and later life health in children, indicating a clear intergenerational impact of the disadvantage that needs to be stopped. It also indicates a positive role for increased education level in health outcomes, and a negative role for adverse childhood experiences which are particularly related to subsequent poor health behavior. This strongly suggests tackling disadvantage to improve health behaviors and reduce ACE.

*Lifepath* researchers also investigated some experimental and non-experimental approaches to mitigate the impact of socio-economic deprivation, and their findings suggest that policy tools (such as conditional cash transfer) are available to reduce the experience of adverse socioeconomic conditions in early life, which have measurable effects on health and economic resources in adulthood. However, the impact of social policies is not always predictable, which highlights the need to evaluate the impact of policies before they are scaled up.

Our statistical modeling suggests that trajectories toward poor health can be modified by acting jointly both on intermediate risk factors and on social deprivation itself. What also emerges from different streams of research is that prevention and mitigation policies must be adapted to contexts, i.e., there is no single intervention model that fits all populations.

## The Underlying Theory

### What Is Socio-Economic Position and How It Is Measured

Research on the social determinants of health is severely hampered by the use of poorly defined and understood social position measures. The Registrar-General's Social Class scheme was the measure used in the classic British data on health inequality between the 1920s and 1991. This series was the basis for the current research on health inequalities, and it is also the basic explanation for current confusions in the research. Understanding some of this history is very helpful in attempting to clarify some of the confusions that surround the measurement of social position.

**Figure 4:**
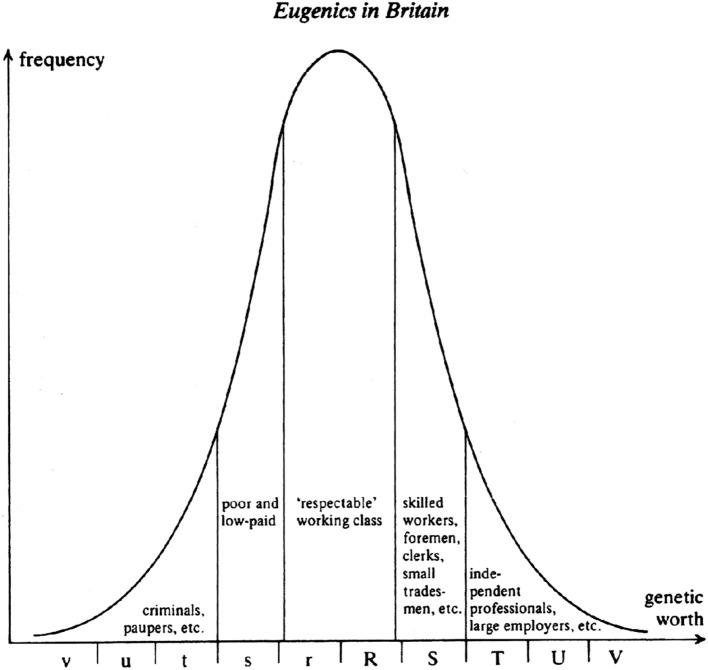
Galton's “Eugenics in Britain”.

Marshall et al., have described the Registrar-General's social class schema in the following way:

“The scheme embodies the now obsolete and discredited conceptual model of the nineteenth century eugenicists: namely, that of society as a hierarchy of inherited natural abilities, these being reflected in the skill level of different occupations” (47). Figure 4 shows Galton's representation of social inequality in the early twentieth century.

This figure is part of a debate on the reasons for the persistence of poor health conditions of the urban working class, even after the implementation of major “sanitary reforms” such as systems of sewage disposal and clean water. Galton and the Eugenics school believed that the reason why health had not improved more among the working class and the poor was because improved sanitation allowed “less genetically fit” babies to survive and contribute to a higher mortality than expected in adults. THC Stevenson, a leading public health official at the time, disagreed. He developed a social class scheme which we would now recognize as the Registrar General's classification in order to test the eugenicists beliefs (Table 2). The original R-G scheme looked like this:

**Table 2:** Classification of Registrar-General's Social Classes (RGSCs) of 1931.

**Class number**	**Description**
I	Professional
II	Managerial
III	Routine non-manual and skilled manual
IV	Semi-skilled manual
V	Unskilled manual

The five classes of Stevenson's scheme came to be elided with the 5 divisions in Galton's distribution of “genetic worth,” despite the fact that Stevenson was not a eugenicist. His work actually found no evidence of a major contribution of the survival of “genetically unfit” babies to the high mortality of adult workers.

The term “socioeconomic status” is often used in health research, but it contains a basic confusion between concepts of class, status, income, and wealth. In epidemiology (though not in social sciences) education is also used as a measure of position in the social structure. To make things worse, these different measures may be used as if they were interchangeable. A few studies such as that of Geyer et al. (48) have tested the validity of this assumption. They found that in fact education, income, and an occupational measure of social class were only moderately correlated, and had different strengths of relationship with different health outcomes.

The use of a particular measure of social inequality defines the mechanisms that are being tested. For this reason, the literature often appears to be inconsistent when in fact it is not. Both plausible confounders and mediators will differ according to the dimension of social inequality that is being used. So we may expect the influence of statistical adjustments to vary among studies, reflecting not opposing evidence but different pathways. And of course great problems arise for those who try to do any kind of synthetic reviewing.

These problems have been summarized in terms of the difficulties they pose for the construction of “plausible narratives,” another way of expressing the notion of a “causal chain” but expressed in perhaps less scientifically contentious terms. In observational life-course research we use the term “causality” at our peril. However, the kind of causality recognized in clinical trials is not sufficient for our purposes either. The presence of an effect in a randomized trial does not necessarily elucidate the biological mechanisms at work. To make sense of health over the life-course there are many mechanisms to understand at different levels, from social institutions to molecules.

Sociology has traditionally drawn clear distinctions between class and status. But since these dimensions of inequality are often correlated, as they are with income and wealth, it can appear that for descriptive purposes it does not matter which one is used. However, if we understand the conceptual basis of the different measures we will greatly accelerate our efforts of explanation. Once these conceptual issues are clarified, it becomes clear that distinct dimensions of inequality implicate etiological pathways composed of different mixtures of material, psychosocial, cultural, and behavioral factors.

In *Lifepath* we have decided to use the generic wording “socio-economic position” (SEP) and to harmonize data in our database accordingly. Social gradients in health have been consistently found using occupational class, educational level, income, or household financial resources, or parents' SEP (occupation or education), with some differences related to the health outcome investigated.

### Life-Course Model of Healthy Aging

#### Introduction

In *Lifepath* we put forward a definition and recommendations about variable selection that captured the life-course component of the *Lifepath* project. This is the definition we proposed: “*Healthy aging is the optimal state of performance and wellbeing capable for any particular phase of the life-course that can be expected in a society, across all social and cultural groups of a population.”*

The key to *Lifepath*'s unique take on healthy aging is the life-course aspect. Healthy aging across all social groups will never be attainable if research focuses only on older adults who are considered to be “aging” or “old.” As an ongoing process, *what aging is* at different points along the life-course needs to be considered. And whatever *it is*, it must be understood as changeable, perhaps even in constant flux. As well as considering time and human developmental processes (i.e., timing), we need to simultaneously remember that *what aging is* at different stages of life varies across socioeconomic groups. Therefore, to some extent we must accept that measuring healthy aging is like trying to grasp smoke, something ephemeral. As such, our attempts at pinning it down will always be imperfect and reductionist.

#### Healthy Aging Over the Life-Course: Getting to Grip With the Social-to-Biological Processes

A comprehensive and detailed description of “an integrated life-course approach to aging” was described by Kuh et al. (49). They recommend taking into account both function and well-being when examining healthy aging, but also to separate functions into various types, including cognitive, physical, biological etc. Given *Lifepath*'s specific interest in the contribution of biological processes to health and healthy aging, this separation of function types is especially important when trying to understand pathways.

The results from analyses carried out within *Lifepath* come to similar conclusions. They show that the socioeconomic environment is an important risk factor for health from early life and throughout life, and acts in part through its effects on biological mechanisms. When considering social-to-biological processes over the life-course, biological mechanisms broadly fit into those of exogenous (behaviors and exposure to pathogens etc.) and endogenous origin (stress response system including different biological dimensions, from proteins to DNA methylation) (50). In *Lifepath* we have repeatedly come back to discuss and dwell on the idea of embodiment and a possible way of measuring it through measurements such as the allostatic load and epigenetic clocks. We will present these issues below, and use the concept of adaptive allostasis to formulate a life-course model of healthy aging.

#### Aging, Adaptation and Adaptive Allostasis

Let us go back momentarily to thinking about aging. Maklakov et al. (51) state the evolutionary “problem” of aging as such:

“Aging is deleterious for Darwinian fitness, yet is a pervasive feature of most living beings. Given the large number of known repair mechanisms, it is not clear why organisms should senesce. This apparent paradox is resolved by the evolutionary theory of aging, which relies on the fundamental principle that the strength of natural selection declines with age, because of extrinsic (non-aging-related) mortality resulting from the cumulative effects of a variety of biotic and abiotic factors” (51).

This highlights the cross-species nature of aging, and its relationship with the theory of evolution. The challenges of living each day result in a depletion of biological resources to restore organisms to full function. The authors explain it thus:

“Hence, the intrinsic organismal repair mechanisms are imperfect, because even if they perfectly repaired all damage, their benefit would gradually be nullified by the increasing risk that the organism will die from other causes anyway.” (51)

So, the nature of life itself leads to physical decline, and all living things are faced with this reality of aging. How to live in the best possible state of health while one's biological organs and systems gradually decline is what we mean by healthy aging.

Adaptive strategies to maximize fitness and survival occur from conception onwards. Published research on the developmental origins of adult health and disease contains numerous examples of adaptive responses to external circumstances. These are proxies of biological aging, occurring very early in the maturation process. Evidence coming from animal studies supports the hypothesis that a biological event affecting a critical period of animal development can permanently “program” the organism. A working definition of this notion of *programming* has been proposed by Lucas, i.e., “an early stimulus or insult, operating at a critical or sensitive period, results in permanent or long-term change in the structure or function of the organism” (52). A set of adaptations, such as small body size and a modified metabolism occurring in early life that would improve an individual's survival in a harsh environment, have been termed the “thrifty phenotype” (53).

By being “programmed” an organism responds to stimuli from the environment by optimally adapting itself to the surrounding conditions, thus improving fitness and prolonging survival in that context. However, such adaptation is beneficial only in the short-term, since, by fixing adaptation so early, the organism is adapted to a specific set of circumstances, but not necessarily if those circumstances change. In terms of the fetal origins of adult disease hypothesis, taking the example of nutrition as an exposure, this implies: (i) programming due to nutritional impairment in early life, and (ii) an abundant and affluent environment subsequent to the initial adaptation. As confirmed by Leon “it may be that nutritional impairment *in utero* (…) coupled with the development of energy dense and westernized diet and life style in adult life could lead to particularly adverse health trends” (54).

Our environment is highly variable requiring a permanent adaptation of physiological systems. Such adaptation through changes is crucial for survival and refers to allostasis (55). Multiple physiological systems, including nervous, endocrine, and immune, are involved in the allostasis processes, all of which come to maturation during the postnatal period into adulthood. Over time, this “adaptive allostasis” allowing us to respond to environmental challenges elicits benefits and costs to the individual. The cost may be minimal, but if environmental challenges change or vary and require continual adaptation, costs may build-up. When the environmental challenges affect socially defined subgroups of the population, the biological cost will be observed at the group level. This “cost” is measurable to some extent biologically, using different approaches.

#### The Cost of Adaptation: Inflammaging, Allostatic Load, and Epigenetic Aging

Chronic exposure to psychosocial stressors, but also interindividual differences in the susceptibility to stress, are associated with a prolonged activation of allostatic systems. This may lead to an allostatic overload with potentially detrimental health consequences. Allostatic load (AL) is therefore the price paid by the body over time for adapting to challenges. It refers to the concept of biological multisystem wastage, whereby “the strain on the body produced by repeated ups and downs of physiologic response, as well as by the elevated activity of physiologic systems under challenge, and the changes in metabolism and the impact of wear and tear on a number of organs and tissues, can predispose the organism to disease” (56).

An AL score should, by definition, be a composite measure including various physiological systems in order to capture overall physiological wear-and-tear. The MacArthur Study of Successful Aging was the first to propose an AL score (57). Parameters included systolic and diastolic blood pressure (indexes of cardiovascular activity); waist-hip ratio (an index of more long-term levels of metabolism and adipose tissue deposition, thought to be influenced by increased glucocorticoid activity); serum high-density lipoprotein (HDL) and total cholesterol levels (indexes of long-term atherosclerotic risk); blood plasma levels of total glycosylated hemoglobin (an integrated measure of glucose metabolism during a period of several days); serum dehydroepiandrosterone sulfate (DHEA-S) (a functional HPA axis antagonist); 12-h urinary cortisol excretion (an integrated measure of 12-h HPA axis activity); 12-h urinary norepinephrine and epinephrine excretion levels (integrated indexes of 12-h sympathetic nervous system activity). Some variants of the original items can be found in the literature, but the markers most commonly used are associated with cardiovascular and metabolic diseases (blood pressure, heart rate, blood glucose, insulin, blood lipids, body mass index, or waist circumference), HPA axis (cortisol, DHEA-S), sympathetic nervous system (epinephrine, norepinephrine, dopamine), and inflammation (C-reactive protein, IL-6) (58).

These scores of AL have been shown to be a better predictor of mortality and functional limitations than the metabolic syndrome or any of the individual components used to measure AL when analyzed separately (59). AL score is also associated with an increased incidence of cardiovascular disease, and poorer cognitive function (54). Recent research also suggests a link between early environment and AL (60–62). As a measure of the global cost of adapting to (and coping with) the environment, AL may be a relevant tool or concept for measuring biological health, and therefore healthy aging. Work on “weathering,” i.e., early health deterioration, as measured across biological indicators of repeated exposure and adaptation to stressors, has been conducted by Geronimus and colleagues among Black communities in the US, suggesting an effect of racial disparities (63, 64).

Epigenetics, specifically DNA methylation modifications, has been proposed as a biomarker of biological aging and as one of the plausible mechanisms through which social exposures become biologically embodied, affecting physiological systems and cellular pathways leading to disease susceptibility (37). The “epigenetic clock” is one of the main mechanisms contributing to age-related methylation changes (65). It refers to specific sites on the genome where methylation levels constantly change as the body ages and can therefore be used to predict chronological age with high accuracy (7). This type of clock can identify deviations between the epigenetic clock and chronological age that may be driven by social exposures. It means that the biological aging of one social group can be compared to another, a useful tool when examining the socially driven differences in healthy aging.

In Lifepath, we have taken an interest in capturing the overall cost of biological adaptive functioning through concepts such as AL, but also other ones that may intercept more specific aspects, such as epigenetic mechanisms or inflammation. A wide literature refers to wide-ranging associations between markers of inflammation and many pathological processes leading to premature morbidity and mortality. As such, the term “inflammaging” has emerged, referring to the role of the inflammatory system in aging processes (66).

One of the big advantages of using biomarkers in health research is that they offer the opportunity to capture a wide range of processes underlying health states. Pathological conditions may be identified, but pre-disease and “normal” or “optimal” biological processes may be measured as well. This ultimately allows us to question what is “normal,” how our biology works under optimal conditions, and the early stages of biological deterioration. Measures such as allostatic load and epigenetic clocks have been explored in the Lifepath analyses, as we will see below.

#### A Life-Course Model of Healthy Aging

In our previous work we used an interdisciplinary method in which we developed a conceptual model of embodiment over the life-course (50). Here we will reformulate this model in relation to the social-to-biological processes involved in healthy aging.

In *Lifepath* we identified the following entities as being required starting points to be outlined in all project proposals: *social and/or psychosocial exposures; outcomes* measuring biology and/or health; *hypothesized mechanisms or pathways*, and *timing or life stage*.

a) Social and/or psychosocial exposures. In *Lifepath*, our research questions try to understand how exposure to socio-material and/or psychosocial conditions may affect health. These variables may therefore take different forms. We largely used proxy variables for the concept of socioeconomic position, including occupation, education, or income. A main issue is to disentangle what exposures/mechanisms are acting behind multidimensional socioeconomic measures such as education, occupation, or income. In *Lifepath*, we took an interest in stressful conditions that may be associated with social position measured using variables capturing adverse childhood conditions, and measures of socio-economic hardship, such as exposure to the great recession, or material deprivation.

b) Outcomes measuring biology and/or health. In *Lifepath* analyses we theoretically wanted to capture either an adaptive response, or a biological cost. To do this, we considered physiological biomarkers to build Allostatic Load measurements, omics markers to measure epigenetic aging, individual biomarkers to examine specific biological processes. However, we also measured health using proxy outcomes such as mortality and different aspects of physical functioning.

c) Hypothesized mechanisms and pathways. These take many possible forms, and in an interdisciplinary project could be countless, given disciplinary specificities. However, a key issue in *Lifepath* is the importance of identifying at least one biological mechanism.

d) Time and timing: socially constructed life stages and human development. The timing or life stage is important, given the longitudinal nature of the data, and of the research questions. The concept of timing leads to exposures or mechanisms taking on importance or being unimportant at a given stage. The concept of timing is particularly important if we consider that social and biological elements change so rapidly over time in the first two decades, but also toward the end of life.

**Figure 5:**
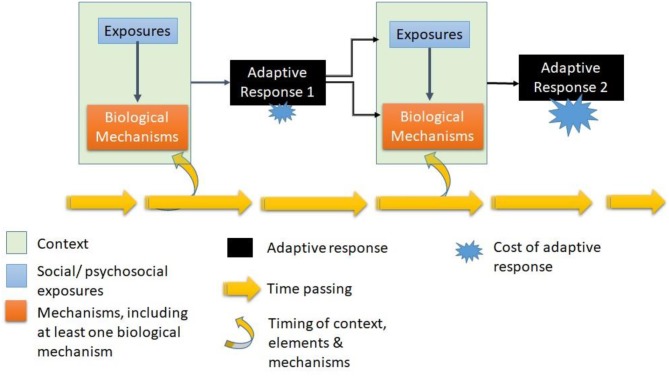
The cost of living: Life-course model of healthy aging (Kelly-Irving, unpublished).

These constituents—exposures, mechanisms, timing—form the life-course model of healthy aging shown in Figure 5. In this model each component is represented, relationships between components are expressed either as interactions (x) or as possible causal pathways. Time is a key component affecting both types of relationships and the response outcome. The time dynamic is drawn both in terms of timing and the passage of time. The “response” is an adaptive one, being neither positive, nor negative. Each time there is a response, with a cost. This cost accumulates over time, however the amplitude and velocity of this accumulation vary according to all the other components of the model.

The resulting life-course model can be used to break-down the healthy aging process at whatever point of the life-course is being studied. It allows us to understand how past responses have influenced the ones being observed and measured, and in turn, how the observed and measured responses will return into the dynamic and influence the next ones. This may be useful to researchers and health professionals when attempting to specify hypotheses regarding life-course mechanisms, and also in interpreting research findings or the outcomes of interventions. This conceptualization both embraces the complexity of life-course research on health, while offering a simplified model.

This model also integrates the methodological challenges of life-course research on health. An isolated element or mechanism being studied as an “exposure” or “mediator” using a proxy variable at a given time may represent a different set of elements and mechanisms depending on the measure of the outcome studied, the life-course stages involved, and the variables included into a statistical model. For example, an outcome used in one set of analyses (e.g., body mass index in childhood), may be a relevant exposure or mediator in a series of future analyses where a different outcome is used (e.g., inflammation). This model of healthy aging can be useful to researchers because it allows us to conceptualize simultaneously the general and highly complex relationship between variables involved in the production of health and health inequalities and to make pragmatic analytical decisions to focus on one segment of the dynamic: specify social exposures; specify mechanisms and notably biological ones; and define the response/outcome of interest. It also allows us to understand how time and timing affect these relationships. What we propose is a tool to facilitate how to think about social-to-biological processes, and to help break down the analysis into meaningful and functional parts.

## Lifepath Results

The following chapters summarize the main results of *Lifepath*. Given the nature of this report we do not describe methods in depth, referring the reader to the original publications. We start with studies in large populations, based on health statistics, followed by studies in cohorts with particular emphasis on mechanisms and biomarker data.

### Health Inequalities at a Population Level

#### Health Inequalities in European Populations and Their Determinants

Data on mortality by socioeconomic position at the population level are not available on a routine basis. The *Lifepath* project collected and harmonized register-based mortality data from a large number of European countries covering all parts of the subcontinent, and harmonized survey data on health and determinants. Based on these unique data, Lifepath conducted: (1) studies on socioeconomic differences in health in European populations using different measures: mortality/life expectancy, disability, and healthy aging (operationalized with disability-free life expectancy), and (2) studies on the contribution of different risk factors to socioeconomic inequalities in health.

#### Building a Database on Health Inequalities and Their Determinants

Data on mortality by socioeconomic position at the population level are not available on a routine basis. We have created a large database of mortality and health data to quantify socioeconomic differentials and trends in mortality/life expectancy, morbidity/disability, and disability-free life expectancy in different European countries, and to identify entry-points for policies to reduce these differentials by quantifying the contribution of a number of specific determinants. The collection and harmonization of mortality data by SEP took place in close collaboration with an existing network of researchers and staff of central statistical offices, which has collaborated to compare socioeconomic inequalities in mortality and morbidity in previous European projects (Eurothine, EURO-GBD-SE and DEMETRIQ). In the *Lifepath* project, these data were extended to cover the complete first decade (or more, depending on data availability after the most recent census) of the twenty-first century. The mortality data stem from a longitudinal mortality follow-up after a census and provide information on all-cause mortality and specific causes of death by level of education and for a subset of selected countries also by occupational status, and cover complete national (in some cases: regional) populations. These data have a good spread between North, West, South, and East. The mortality data formed the basis of analyses of socioeconomic differences in mortality and life expectancy and served as input for the calculation of healthy aging measures, such as disability-free life expectancy.

In addition, EU-wide survey data on self-reported morbidity, disability, and determinants by age, sex, and socioeconomic position were derived from large scale international surveys. Survey data on health in combination with mortality data served as input to calculate disability-free life expectancy. Data on determinants served as input to estimate their contribution to inequalities in mortality/life expectancy, disability, and disability free life expectancy.

#### Health Inequalities in European Populations

As starting points for analyses on trends in inequalities and for analyses on the contribution of determinants, we documented inequalities in all cause-mortality (29), life expectancy (67, 68), mortality from specific causes (69–71), disability (72, 73), and disability-free life expectancy (67) across Europe. This pointed at large inequalities for all health measures, but with important variations between countries and genders. Inequalities were larger in Central & Eastern Europe than in other regions, and were larger in men.

#### SEP and Trends in Mortality and Health in the USA and Europe

**Figure 6:**
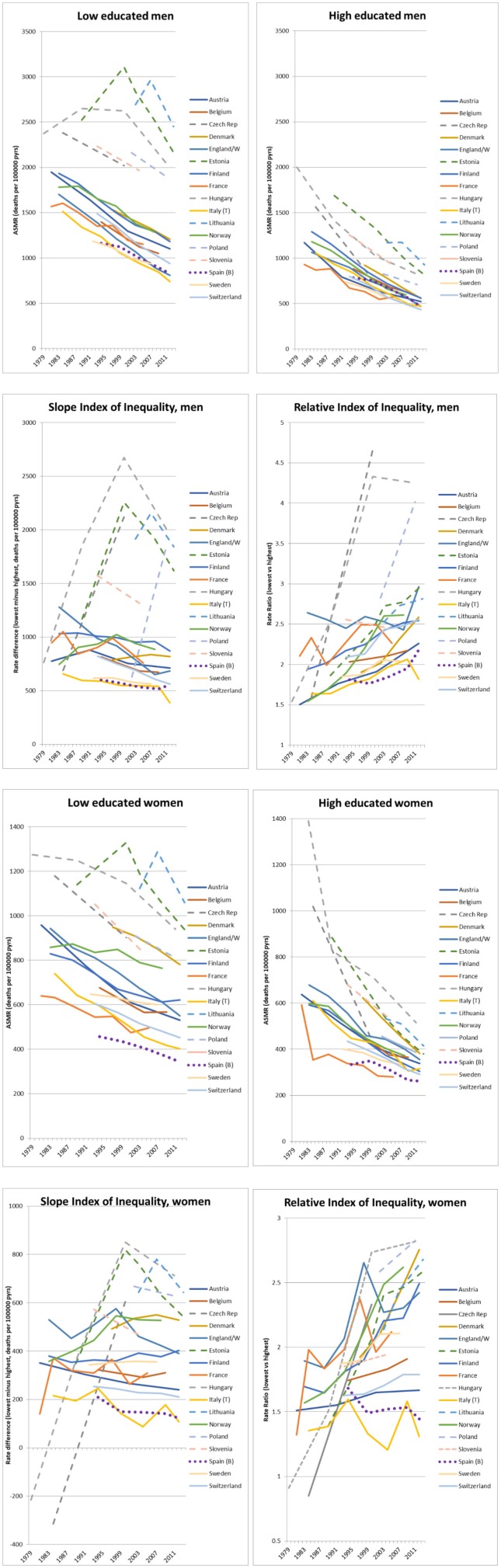
Trends in all-cause mortality by educational attainment, absolute and relative index of inequality, ca. 1980-ca. 2014 (29). Dashed lines: Eastern Europe. Dotted line: Western European countries most severely hit by the economic crisis. Measures of mortality on the y-axis are rate differences, rate ratios or ASMR = age-standardized mortality rate.

Unfavorable health trends among people with disadvantaged SEP have recently been reported from the United States. *Lifepath* (29) analyzed health trends by education in European countries, with particular attention to the possibility of recent trend interruptions, including those related to the impact of the 2008 financial crisis (Figure 6). We collected and harmonized data on mortality (1980 to 2014) for 17 countries including 9.8 million deaths, and information on self-reported morbidity from 2002 to 2014 for 27 countries including 350,000 survey respondents. We used interrupted time series analyses to investigate changes over time and country-fixed effects analyses to investigate the impact of changing economic conditions on health. Recent trends were more favorable than in previous decades, particularly in Eastern Europe; here, mortality started to decline among men with more disadvantaged SEP and the decline in less-than-good self-assessed health accelerated, leading to a moderate narrowing of health inequalities. In Western Europe, mortality has continued to decline among the low and high SEP categories, and although the decline of less-than-good self-assessed health was slower in countries severely hit by the financial crisis, this affected SEP categories equally. Economic conditions related to the crisis were not associated with widening health inequalities. Our results suggest that the unfavorable trends observed in the United States are not detected in Europe. There has also been no evident short-term impact of the crisis on health inequalities at the population level.

#### SEP and Trends in Mortality From Conditions Amenable to Health Care in Europe

As little is known of the effectiveness of health care in reducing inequalities in health, *Lifepath* (70) focused on conditions amenable to health care. This study assessed trends (1980-2010) in educational inequalities in mortality from conditions amenable to health care in 17 European countries, and studied the determinants of these trends using country fixed effects models. The study showed that remarkable declines in mortality from conditions amenable to health care have occurred among both the lower and higher educated, but while absolute inequalities have been largely stable, relative inequalities have risen considerably over the last three decades in the European countries covered by the study. This is due to faster relative mortality declines among the higher educated for most amenable causes, with only few exceptions. Higher health care expenditure was associated with lower mortality from amenable, but not from non-amenable causes. The effect of health care expenditure on amenable mortality was equally strong, in relative terms, among the low and the high educated, and as a result higher health care expenditure was associated with a narrowing of absolute inequalities in amenable mortality.

#### SEP and Trends in Mortality From Cardiovascular Diseases in Europe

Cardiovascular diseases (CVD) are still one of the leading causes of death in Europe and mortality from CVD is known to be unequally distributed across socioeconomic groups. *Lifepath* (69) assessed if recent declines in cardiovascular mortality led to benefits in all socioeconomic groups evenly and if these declines have narrowed or widened inequalities in mortality from cardiovascular disease (CVD). The study showed that in the early 2010s, educational inequalities in CVD mortality were smallest in Southern Europe, intermediate in Northern and Western Europe and largest in Central & Eastern European countries. CVD mortality has declined rapidly among lower and higher educational groups, and although relative declines were faster among subjects with higher SEP, absolute declines were almost uniformly larger among lower SEP groups. As a result, relative inequalities increased over time, but absolute inequalities often declined substantially. For those populations in which an assessment of occupational inequalities in CVD mortality was possible, the picture was largely similar to that seen for educational inequalities. Men in manual occupations experienced the largest absolute declines in mortality whereas those in upper non-manual occupations experienced the largest relative declines. The geographical divide seen for educational inequalities in CVD mortality was apparent also for occupational inequalities.

#### SEP and Trends in Mortality in 8 European Countries, Japan, and South Korea

Inequalities in mortality in Japan and South Korea have been reported to follow different patterns from those in other high-income countries, but systematic comparisons have not been performed. *Lifepath* (71), therefore, investigated mortality inequalities by occupational groups in Japan and South Korea with those in 8 European countries. National register-based data from Japan, South Korea, and 8 European countries [Finland, Denmark, England/Wales, France, Switzerland, Italy (Turin), Estonia, Lithuania] covering the period between 1990 and 2015 were collected and harmonized. We calculated age-standardized all-cause and cause-specific mortality among men aged 35–64 by occupational class and measured the magnitude of inequality with the Average Inter-group Difference (absolute and relative version). Clear gradients in mortality were found in all European countries throughout the study period: manual workers had 1.2–3.6 times higher mortality than upper non-manual workers. However, in the most recent time-period, upper non-manual workers had higher mortality than manual workers in Japan and South Korea. This irregular pattern emerged as a result of a rise in mortality among the upper non-manual group in Japan during the late 1990s, and in South Korea during the late 2000s, due to rising mortality from cancer and external causes (including suicide). In conclusion, patterns of mortality by occupational group are considerably different between European countries and Japan and South Korea. The irregular patterns in the latter two countries are probably due to a larger impact on the higher occupational groups of the economic crisis of the late 1990s and the late 2000s, respectively.

#### Determinants of Socioeconomic Inequalities Health in Europe

##### SEP and determinants of disability

Previous studies have shown the existence of social inequalities in disability in many European countries. However, it is not clear what factors are associated with these inequalities. *Lifepath* (72) assessed the contribution of behavioral factors, occupational factors, and living conditions to educational inequalities in disability. We pooled data from the seventh wave of the European Social Survey (ESS) (2014) which included self-reported disability measured with the Global Activity Limitations Indicator (GALI) for 19 European countries. We used data from respondents aged 30–79. The prevalence of disability was higher among women and in the low educated in almost all countries, but the magnitude of these inequalities differed substantially between countries. In the pooled dataset, occupational factors were the most important contributor among men, and behavioral factors among women, but there were large variations between countries in the contribution of determinants. Inequalities in disability are a major challenge for public health in most European countries. Our findings suggest that these inequalities can be reduced by diminishing inequalities in exposure to determinants such as occupational and behavioral factors.

##### SEP and determinants of inequalities in life expectancy

Socioeconomic inequalities in life expectancy have been found in all European countries, but it is not known what the most important determinants are. *Lifepath* (68) quantified the contribution to inequalities in life expectancy of eight risk factors for mortality. We collected mortality data and survey data on father's occupation, income, social contacts, smoking, high alcohol consumption, body-weight, physical exercise, and fruit & vegetable consumption. For each country, we estimated inequalities in life expectancy, and determined the effect of changing the prevalence of each risk factor among the low educated to that of the high educated (“upward leveling scenario”), using a method based on Population Attributable Fractions. The setting included 15 European countries, with 2.7 million deaths occurring in 294 million person-years. The main measures of outcome were partial life expectancies between the ages of 35 and 80 by level of education. In all European countries, there was a substantial gap in life expectancy between low and high SEP, of between 2.3 and 8.2 years among men and between 0.6 and 4.5 years among women. The risk factors contributing most to the gap in life expectancy were smoking (19.8% among men, 18.9% among women), low income (9.7 and 13.4%), and high body-weight (7.7 and 11.7%), but there were important differences between countries in the contribution of different risk factors. However, sensitivity analyses using the prevalence of risk factors in the most favorable country (“best practice scenario”) showed that the potential for reducing the gap may be considerably smaller. The results were also sensitive to variable assumptions about the mortality risks associated with each risk factor.

In conclusion, smoking, low income and high body-weight are important entry-points for policies to reduce the impact of educational inequalities on life expectancy in most European countries, but priorities differ between countries. Our results provide an upper limit to what can be achieved by policy action to mitigate the social distribution of single risk factors, but it is clear that a substantial reduction of inequalities in life expectancy requires strong policy actions on a broad spectrum of health determinants.

##### SEP and determinants of inequalities in disability-free life expectancy

One modifiable risk factor associated with an increased risk of both mortality and disability is low fruit and vegetable consumption. It has been established as a risk factor for all-cause mortality, with pathways via cardiovascular diseases, cancer, and other, yet unspecified diseases causing increased mortality rates. Fruit and vegetable consumption varies between educational groups across Europe; a higher level of education is overall associated with a higher consumption of fruit and vegetables. *Lifepath* (67) quantified the contribution to educational inequalities in life expectancy of fruit and vegetable consumption in 10 countries. For this purpose, mortality data by age, sex, and level of education were obtained for each country from national census or registries with mortality follow-up including at least data on years 2010 or later, where available. Where no follow-up data were available, we used cross-sectional data provided by the respective countries. We included data for ages 35 to 79 years, excluding age 80 and over, since data on mortality by educational level are less reliable in this category. Data on disability prevalence were obtained from the European Union Statistics on Income and Living Conditions (EU-SILC), years 2010 and 2014, and data on prevalence of low fruit and vegetables by sex, age, educational level, and country from round 7 (2014) of European Social Survey (ESS). Our analyses show that improving consumption of fruit and vegetables in low educated groups to the level of high educated would have a small, but positive effect on disability-free life expectancy (DFLE), and has the potential to reduce inequalities in health; in particular in countries where inequalities in disability-free life expectancy and fruit and vegetable consumption are large. In more than half of the assessed countries, 50% or more of the potential effect of increasing fruit and vegetable consumption could be achieved by upward leveling.

A new *Lifepath* study (67) extended these analyses to include 8 risk factors and 16 countries. Low income is the risk factor that contributes most to the disability-free life expectancy gap between educational levels. This is followed by smoking, father's manual occupation and high body weight; however, there are important differences between European countries in the relative importance of risk factors.

## Health Inequalities in Cohorts and the Underlying Mechanisms

### Background

While the field of social epidemiology has produced solid evidence of a social gradient in health and quality of aging, questions persist as to (i) how this gradient depends on local and contextual factors (e.g., how global and general such observations are), (ii) which compartments of health status are primarily affected, (iii) which physiological systems are involved in the response to social adversity and how these impact health in the life-course, (iv) what molecular mechanisms are triggered by different social exposures. In order to address these high priority research questions, *Lifepath* leveraged the large diversity of complementary data sets including:

- Cohort data including hundreds of thousands of individuals with detailed anthropometric, lifestyle, and social factors, as well as low-resolution clinical outcomes.
- Sub-cohorts including thousands of individuals in whom biosamples were collected and used to measure either targeted biomarkers or full resolution omics profiles.
- Data featuring repeated measurements of clinical variables, and/or biomarkers, and/or omics profiles.

To integrate this information and to address our research questions we adopted an integrated analytical framework first exploring the links between social factors and health. This relied on combining large (up to millions of individuals) and heterogeneous data sets and required to account for the role of age and the existence of potential critical life stages at which social factors preferentially exert their effect via the implementation of lifecourse models. A second stream of analyses focused on the investigation of the markers, and potential mechanisms, involved in the embodiment of social experiences. As detailed in the second section of the present chapter, these analyses encompassed different levels of resolution from low-resolution synthetic scores such as the Allostatic Load to full resolution omics profiling. These analyses provided different balances between data resolution and result interpretability and provided complementary information on how social experiences were biologically embodied. A third stream of analyses related the biological marks of social experiences and health outcomes. These analyses needed to account for timing of exposures and biological effects.

All the results shown below are from published *Lifepath* papers.

#### Lifepath Evidence of Social Gradients in Health and Quality of Aging

While the existence of social gradients in health is now established, there were still inconclusive observations regarding the generalizability of these gradients, and uncertainties with regard to the role of local and contextual factors. To investigate these aspects of the effects of social experiences on health, Stringhini et al. combined data from 48 cohorts covering 1.7 million individuals from seven high-income countries and investigated the association between occupational position and mortality (7). These analyses identified a systematic gradient in mortality across countries in both males and females leading to higher all-cause mortality for more disadvantaged occupational groups. This association remained statistically significant upon adjustment for the WHO 25x25 factors (including alcohol, smoking, physical activity, hypertension, obesity) and resulted in an overall 2 years shorter life expectancy for individuals within the more disadvantaged occupational group compared to the less disadvantaged. This change in life expectancy was found similar to that induced by physical inactivity and hypertension, and greater than the one induced by obesity and high alcohol intake. Overall this study showed that there was a significant effect of social adversity during adulthood on health, as measured by all-cause mortality, and that these adverse effects were (i) not fully driven by established health risk factors, and (ii) consistent across high-income countries populations.

A subsequent set of analyses pooled data from 37 cohorts, totaling over 108,000 participants from 24 countries in Europe, the US, Latin America, Africa, and Asia, and investigated the association between socio-economic position (as measured by occupation) and functional outcomes as measured by walking speed (8). As a high priority aim of *Lifepath*, this work focused on the number of years of functioning lost (YFL), which were estimated for different age groups, and its association with occupation. Results showed a consistently significant number of years of functioning lost across countries. In high-income countries, the number of YFL exceeded 8 years for men and 5.4 years for women while comparing low and high occupational groups at the age of 60, and the effect of social adversity appeared stronger in the US than Europe. In low- and middle-income countries, this loss was reduced in both men and women.

In both studies the effect of social factors on health and aging was robust to the adjustment for established health risk factors, but the contribution of the latter, including behaviors, in social gradients for health remains unclear. Our systematic review included over 100 publications to quantify the evidence linking unhealthy behaviors to their health consequences (74). This work has provided evidence that unhealthy behaviors (and in particular smoking) explain a larger proportion of health differentials than SEP itself, but suggested that this contribution was heterogeneous across health outcomes (greater for all-cause mortality), SEP-indicator, region, gender, and age.

While this work confirmed the role of behaviors in health, it also suggested that the role of behaviors was complementary or at least not fully overlapping with that of socioeconomic position, in the determination of the quality of aging. This work also evidenced the importance of the social context, which seems to act as an effect modifier.

This highlighted the need for refining and adapting the socio-economic-related exposures to the system and context they relate to. A good example of that approach involves social differences in the risk of infection by Epstein Barr virus (EBV) in children (*N* > 12,000) from the Millennium Cohort Study (25). Authors showed that children from disadvantaged social background were more likely to be infected by EBV, by the age of 3 compared to advantaged children, due to the material conditions to which they were exposed to. In these analyses, social exposures were refined and included environmental factors, and household environment (e.g., temperature in baby's room). The outcome of interest, the EBV infection, is usually benign, but the time of infection can be socially patterned. It was therefore used as a proxy for potential socially-driven differential immune maturation and function which can, later-in-life, affect health.

One major limitation of most of the aforementioned studies resides in the fact that they were usually based on mature cohort data. Recently, we investigated the differences in socially-patterned risk factors from childhood to middle-age using data form the young Finns study (10). This study included more than 3,000 participants followed-up in time on eight occasions, and investigated the changes in 10 risk factors for children from disadvantaged and advantaged neighborhoods. This study showed that social adversity manifested early in life with (i) differential diets (e.g., lower fruit intake at 6 in disadvantaged neighborhoods), (ii) lower physical activity and increased obesity at 12, and resulted in higher prevalence of (pre-)clinical conditions, such as decreased insulin sensitivity and increased fasting glucose in early adulthood. This work clearly identified the pivotal role of age-specific exposures in the social gradient in health trajectories, and reinforces the need to adopt a life-course approach while analyzing the role of social factors on health.

#### Investigating Social Embodiment in Lifepath

Social embodiment can be defined as the sustainable biological response to social adversity and related chronic stress. It involves the (possibly persistent) dysregulation of several physiological systems. Devising an exposome approach including social factors (7, 8, 75, 76) relies on the characterization of how social experiences are translated into specific biological alterations, using available biomarkers, including high throughput omics profiles (77, 78). In *Lifepath*, we considered three main levels of resolution: (i) low-resolution synthetic scores such as the Allostatic Load (AL), (ii) targeted analyses on prioritized pathways, and (iii) full-resolution (multi-)omic profiles.

#### Using Synthetic Scores in Lifepath

It is now established that social adversity, and in particular in early life, has persistent biological effects that contribute later in life to health inequality (3, 4, 58, 79). Over the past few years, intensive research efforts have produced convincing evidence that the biological embodiment of social adversity involves several physiological systems including inflammation, and more generally those related to the stress response. This has been formalized in the concept of Allostatic Load, which measures, through a composite score, the organism's adaptive cost to stressful conditions encountered across the life-course. Recent developments related to the allostatic load have shown that socioeconomic adversity, particularly in early life, leads to a higher load (that is increased life-long stimulation of several key physiological systems), which has been related to increased risk of health outcomes including mortality, morbidity and unhealthy aging. Building upon this research, the Allostatic Load has been at the center of several investigations in *Lifepath*.

For example, the SKIPOGH study (*N* = 1,128 participants in Switzerland) provides data for 14 blood-derived biomarkers, covering six physiological systems (Cardiovascular, Metabolic, Lipidic, HPA, Oxidation, and Inflammation) (80). In this study, the relationships between AL and social factors were investigated (i) for two different markers of social factors: education as a proxy for early life socio-economic position, and occupational position as a proxy for adulthood SEP, and (ii) in men and women separately. Results showed a systematic higher load (i.e., higher level of physiological wear-and-tear) in disadvantaged social groups. Comparing results across SEP-markers and in both genders, it appeared that differences in the AL were stronger, and statistically significant, in relation to education, and in women. In addition, adjustment for behaviors only marginally attenuated the associations. Altogether this work highlighted a social gradient of AL which could not be fully explained by usual socially patterned exposures/behaviors.

In order to disentangle the possible mediating roles of these different time-resolved social experiences, the authors conducted path analyses to estimate the relative contribution of parental SEP factors and own education to the individual AL.

**Figure 7:**
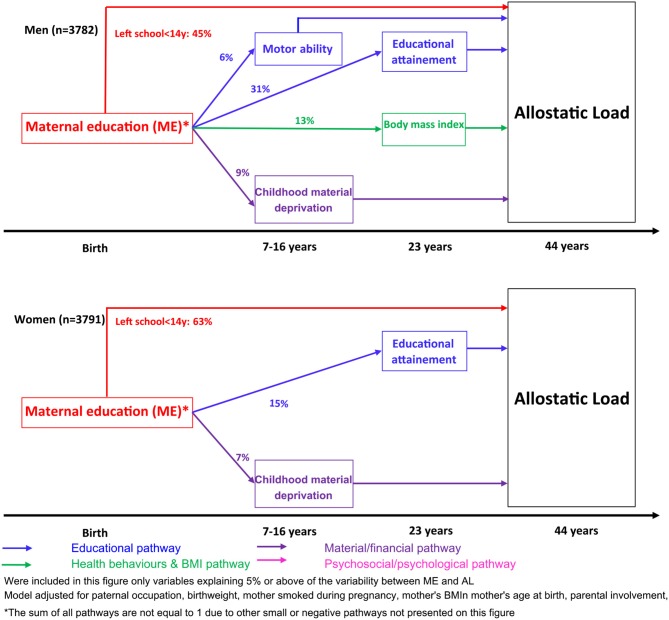
Path Analyses investigation of the relative contribution of different SEP factors to the individual AL (6).

This has been formalized into the direct acyclic graph (DAG) presented in Figure 7 and clearly highlighted a primary role of maternal education in the construction of individual AL later in life (6). Mediation analyses also suggested that own educational attainment was mediating the effect of maternal education on own AL.

These papers have provided solid evidence that early life adversity was a key driver of later-in-life physiological status.

In order to further investigate the timing of social embodiment, we used data from Understanding Society (9,088 participants aged 20–80 years old), on 16 blood derived biomarkers capturing 6 physiological systems (including cardiovascular, inflammation, metabolic, endocrine systems and the functions of both liver and kidney). We (i) defined the Biological Health Score (BHS) as an extension of the AL incorporating two additional systems, (ii) investigated potential social gradients in BHS, (iii) explored the relative contribution of each physiological system to the BHS, and (iv) explored potential age differentials in BHS (46).

**Figure 8:**
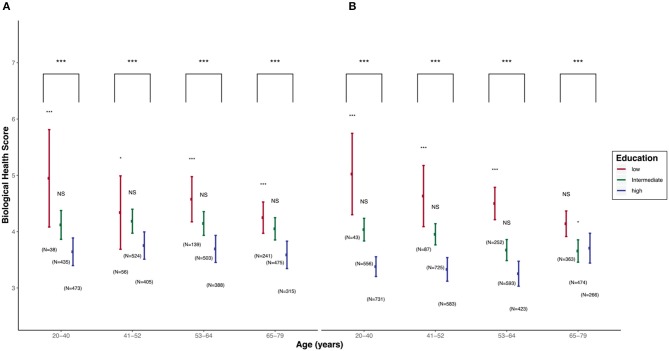
Distribution of the Biological Health Score (BHS) in Understanding Society for Men **(A)** and Women **(B)** by age class (46). *p*-values were coded as * for *p*-values in [0.05, 0.01], ** for *p*-values in [0.01, 0.001], and *** for *p*-values < 0.001.

As presented in Figure 8, this work showed a strong social gradient in the BHS leading to higher scores (i.e., poorer health) in the more disadvantaged social group (as measured by own education).

Data also supported a linear trend in the BHS across SEP groups. This investigation showed that such gradients survived adjustment on the main socially-driven exposures and behaviors. This work contributed to the *Lifepath* evidence of social embodiment in early life, as social gradients in BHS could be detected as early as in the 20's of Understanding Society's participants. Results also suggested that these gradients could not be fully explained by exposures and behaviors. The analysis of system specific sub-scores showed consistent and important contribution of the inflammation system to the overall score in both genders, across age groups and educational attainment, while some other systems (e.g., metabolic system, kidney function) appeared to have a differential contribution to the score at different ages and for different education groups.

#### Analyses Focusing on Inflammation

**Figure 9:**
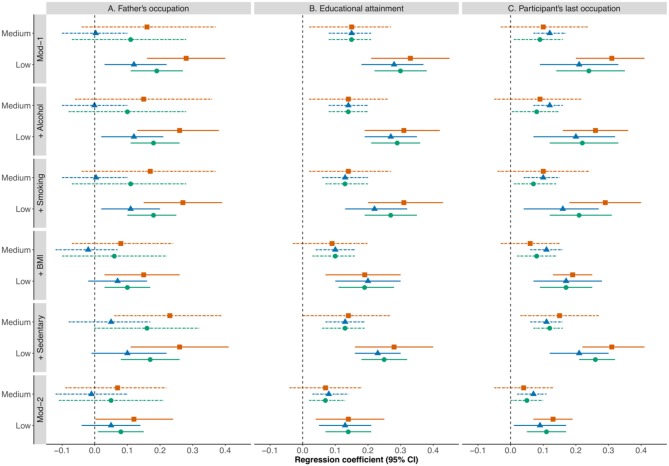
Meta-analysis of the association between CRP and SEP as measured by father's occupational position **(A)**, own education **(B)**, and own occupation **(C)** in the full study population (orange), men (blue), and women (green). Several models are considered and are gradually adjusted for exposures and behaviors (36).

Most of the analyses that were conducted indicated inflammation as a primary and pivotal system in the embodiment of social adversity. To get a better insight into the role of inflammation, several studies were conducted, first investigating the association of C-reactive protein as a proxy for systemic inflammation and SEP factors (36). In this study, data from 6 *Lifepath* cohorts combining over 23,000 participants with blood measurements of CRP was used to explore the link between SEP and inflammation over the life-course. CRP is an established marker of low-grade inflammation. As illustrated in Figure 9, a clear gradient in CRP leading to higher inflammatory burden in the lower SEP group was observed irrespective of the SEP metric. Stronger gradients were consistently observed in women, and while some associations were attenuated upon adjustment for potential confounders (e.g., BMI), most remained statistically significant in the fully adjusted model. This work has provided consistent evidence that inflammation status was affected by social experiences from early-life on, and that these differences were detected irrespective of contextual factors as a wealth of heterogeneous studies were included in these meta-analyses.

In order to capture the complexity of the inflammatory response, a set of three pilot studies were then conducted combining inflammatory proteins and additional omics profiles. These analyses were based on data from the EnviroGenoMarkers project, where more than 230 participants were enrolled and in whom we measured in blood (i) targeted proteomic data (*N* = 28 inflammatory markers), (ii) full resolution gene expression data (*N*~30,000 transcripts), and (iii) full resolution DNA-methylation profiles (*N*~450,000 CpG sites). For each participant, one SEP proxy was available for early life (paternal occupation), young adulthood (own education), and later adulthood (own household's highest occupation). In a first publication Castagné et al. investigated the association between all 28 inflammatory proteins (or a composite score combining all proteins), and SEP factors in a life-course model (32). These results showed that father's occupation was associated with an overall higher inflammatory burden later-in-life irrespective of the way inflammation was measured.

This association was found to be statistically significant regardless of subsequent experiences, suggesting a persistent embodiment from early life onwards. These associations were also robust to adjustment for lifestyle and behaviors. This work was followed-up by an investigation using gene expression data available in the same individuals (81). In this paper, authors defined an inflammatory transcriptome score combining 845 inflammation-related transcripts. These analyses yielded the exact same conclusions, suggesting that the early-life embodiment of social adversity could also be detected at the gene expression level and unaffected by later life social experiences.

These analyses were finally completed by investigating the inflammatory score at the DNA-methylation level using 61 cis-acting CpG sites and found similar life-course associations linking higher inflammation and early-life adversity.

Altogether the analyses we have conducted clearly identified inflammation and related mechanisms as being affected, early in life, by social adversity, and provided some insights into the sustainability of these early life marks, and possible mediating sub-pathways. They therefore advocate for the use of higher resolution molecular data to investigate the biological response to social adversity.

#### Higher Resolution Investigations of Social Embodiment

In an attempt to evaluate the information from full-resolution DNA methylation profiles measured at three different time points (birth, 7 years old, and 15 years old), Alfano et al. performed a series of methylome-wide association studies in relation to maternal and paternal education and occupation (40) using ALSPAC data (*N* = 910 followed-up children).

**Figure 10:**
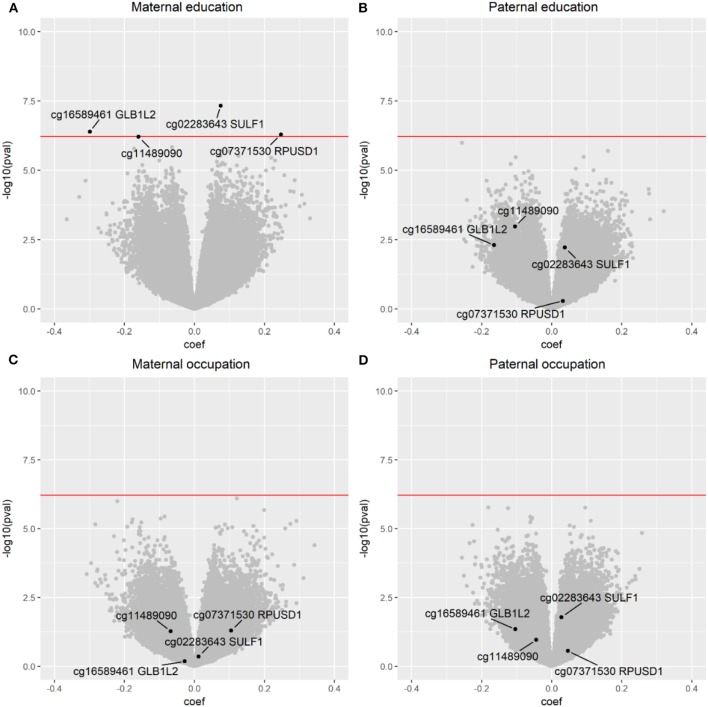
Results from the methylome-wide association study relating cord blood DNA methylation profiles and maternal and paternal education or occupation. **(A)** Maternal education, **(B)** Paternal education, **(C)** Maternal occupation, **(D)** Paternal occupation. Results are derived from the ALSPAC cohort (40). In the “volcano plot” the x axis is related to a measure of the strength of the signal (i.e., association with SEP in this case), and the y axis is related to the statistical significance of the signal. CpGs above the red line are statistically significant after correction for multiple comparisons.

Results showed more associations (i.e., more differentially methylated CpG sites) with maternal education than with other SEP factors (Figure 10).

Associations detected in *cord blood* in relation to maternal education were not detected in relation to other SEP factors. Similar analyses did not detect any differentially methylated CpG sites in relation to maternal education in blood samples from *7 years old*, and found 20 differentially methylated sites in blood samples from *15 years old*. Of these no formal overlap was identified across ages but changes in methylation in the SULF1 gene appeared as a possible common target.

While these results provided interesting proof-of-principle, validation in external studies was not achieved, at least partially due to small sample sizes and resulting insufficient statistical power.

In order to overcome this limitation, several investigations in *Lifepath* used established epigenetic clocks as markers/predictors of accelerated aging and related them to SEP factors. One paper used data from three *Lifepath* cohorts including more than 5,000 participants where full resolution DNA methylation profiles were available (37). From these data Fiorito et al. calculated the epigenetic age of each participant using both Horvath (82) and Hannum (83) epigenetic clocks, and inferred age acceleration as the difference between the biological and chronological age. Irrespective of the way it was calculated, the age acceleration was related to SEP and SEP trajectories. As summarized in Table 3, these analyses suggested a quicker age acceleration (almost 1 year quicker) for the lower education group, and some evidence (except for one of the contributing cohorts) of a linear trend across education groups. Results were only marginally attenuated upon adjustment for behaviors and were similar for other SEP variables. This work also showed that the age acceleration (AA) was less important in individuals experiencing upward social mobility. These results have been confirmed and replicated in a large analysis of 17 cohorts (38).

**Table 3:** Association between age acceleration and Education in 3 LIFEPATH cohorts (37).

**SES**	***N***	**β (95% CI)**	***p***	***I*^**2**^**
EPIC Italy				
High	624	0.00 (reference)	–	
Medium	643	0.82 (0.07, 1.57)	0.03	
Low	514	1.03 (0.29, 1.77)	0.01	
Linear trend	1,781	0.41 (0.09, 0.74)	0.01	
**MCCS**				
High	952	0.00 (reference)	–	
Medium	948	0.46 (−0.31, 1.07)	0.13	
Low	917	0.84 (0.17, 1.51)	0.01	
Linear trend	2,817	0.40 (0.09, 0.71)	0.01	
**TILDA**				
High	168	0.00 (reference)	–	
Medium	158	1.06 (−0.63, 2.75)	0.22	
Low	163	1.03 (−0.72, 2.79)	0.25	
Linear trend	489	0.52 (−0.36, 1.39)	0.25	
**Meta-analysis**				
High	1,744	0.00 (reference)	–	–
Medium	1,749	0.75 (0.17, 1.34)	0.01	0
Low	1, 591	0.99 (0.39, 1.59)	0.001	0
Linear trend	5,087	0.42 (0.15, 0.68)	0.002	0

#### Markers of Social Embodiment and Clinical Outcomes

A third stream of analyses conducted in Lifepath aimed at relating markers of social embodiment and health outcomes. Markers of social embodiment can be envisaged at different levels of granularity and we report in this section Lifepath work referring to (i) composite scores, (ii) (multi)-omic clocks and several health outcomes.

#### Composite Scores and Health in Lifepath

Building upon the work carried out on the AL in the British Birth Cohort, Castagné et al. (84) used the data from more than 8,000 participants to calculate AL using 14 blood-derived biomarkers covering 4 physiological systems: neuroendocrine, inflammation, metabolic, and cardiovascular. These were related to all-cause mortality in order to evaluate the effect of AL on mortality, and to investigate the role of SEP and behaviors in these associations (85).

**Figure 11:**
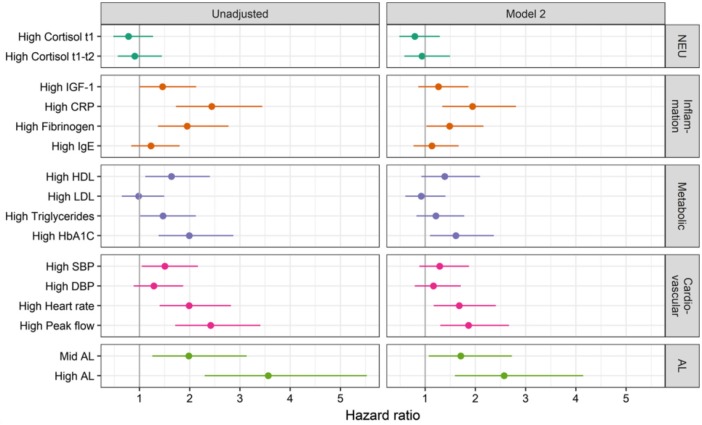
Cox proportional hazard ratio using 14 blood derived biomarkers, or the full AL as predictors of all-cause mortality. Results are presented unadjusted or adjusted for early life, childhood, young adulthood, and adulthood confounders. Results are represented as sex adjusted Hazard ratios (84).

Analyses used either (i) the full AL, (ii) all 14 individual biomarkers as predictors of all-cause mortality (Figure 11) in a crude model and one model adjusting on behaviors.

Results indicate for each system except the neuro-endocrine, a clear and consistent contribution of all biomarkers to mortality, i.e., higher levels of these biomarkers increased mortality. Attenuation of these effects was observed upon adjustment for confounders. However, in the Inflammatory, Metabolic, and Cardiovascular systems, at least one biomarker remained significantly associated to mortality in the fully adjusted model. Interestingly, strongest results were observed for the full AL. This suggests that the AL is able to capture the complementary mortality-relevant information brought about by each separate system. Overall this work has shown that AL calculated at 44 years of age was able to predict mortality regardless of subsequent socio-economic experiences, and that AL as a multi-system composite score was a better and more robust predictor than each of the systems separately.

#### Biological Age and Health Outcomes in Lifepath

Using data from the Melbourne Collaborative Cohort Study (MCCS) including 2,818 participants in whom full resolution DNA methylation data were available, Dugué et al. investigated the association between Horvath and Hannum age acceleration metrics and mortality and evaluated the role of major health risk factors in these associations (39). Four models were considered and were gradually adjusted on different sets of established health risk factors.

Results suggested a significant association between age acceleration (AA) and mortality irrespective of the method used to infer AA. AA derived from Hannum clock was no longer significant upon adjustment on lifestyle, behaviors, socio-economic factors, and clinical variables, while AA derived from Horvath clock was unaffected by these adjustments. This suggests that these clocks (and inferred AA) are capturing different aspects of age acceleration, and that Horvath-AA focuses more on features of aging that are not directly linked to established risk factors. Nevertheless, both measures of AA were found to be associated with major health risk factors. Altogether, this work suggested that AA captures features of biological aging that are relevant to mortality and that different ways of calculating AA do not capture the same health-relevant information.

#### Conclusions and Perspectives

One of the main goals of *Lifepath* was to develop and apply appropriate statistical approaches to identify social gradients in health, to explore if and how social experiences in the life-course get embodied, and finally to investigate the effect of the biological responses to social experiences in relation to health outcomes and quality of aging. Such objectives involved the analysis and combination of complex and heterogeneous sets of data featuring large gradients of size (from hundreds to millions of individuals) and corresponding inverse gradients of granularity and resolution (from a few to hundreds of thousands of variables). A diverse set of methods was to be considered and borrowed from the fields of population sciences, life-course epidemiology, and omics profiling and integration. In particular, we used (and developed novel) synthetic scores such as the AL and the BHS or established markers of biological aging (e.g., DNA methylation clocks). As an alternative, we focused on prioritized biological pathways (e.g., inflammation). We subsequently related these synthetic variables to social factors to investigate whether these were involved in social embodiment, and to health outcomes to evaluate their contribution to (potentially accelerated) aging and disease onset.

Based on these approaches, *Lifepath* has produced solid and reproducible evidence of a SEP-related health gradient for individuals in disadvantaged environments to suffer from poorer health. This result has been shown (i) not to depend on contextual factors (i.e., being generalizable across countries), (ii) to be valid for low-resolution outcomes such as mortality, but also functional outcomes, (iii) to be detectable for more specific outcomes (e.g., cardiovascular health).

*Lifepath* also produced strong evidence for the existence of social embodiment. More specifically, we showed that it could be captured at different levels of resolution: synthetic scores (AL and BHS), accelerated aging metrics, and molecular data (e.g., DNA methylation). The use of composite scores appeared to provide a good balance between interpretability, granularity, and reproducibility. Throughout the analyses performed in *Lifepath*, inflammation appeared as a central system involved in both social embodiment and the development of sub-clinical and clinical conditions leading to accelerated aging. We showed that other pathways/physiological systems (e.g., the metabolic system) were also involved in social embodiment. In many of our analyses, early life appeared as a critical life stage at which social adversity is persistently embodied. Finally, our work provided proof-of-principle results supporting the use of multi-omic data in relation to prioritized scores, or pathways, to identify potential (sets of) omics markers functionally involved in the physiological dysregulation triggered by social adversity and affecting future disease risk or mortality.

To further assess the resulting mechanistic hypotheses, causal models can be developed. As a pilot example, Laine et al. (86) used data from 7 *Lifepath* cohorts (*N* > 170,000) to quantify the (direct and indirect) effect of modifiable risk factors (smoking, alcohol intake, dietary patterns, physical activity, body mass index, hypertension, diabetes, and coronary artery disease) on SEP-related mortality. Using counterfactual natural effect models, the authors have shown that up to 34–38% of the effect of education on mortality was driven by modifiable risk factors, hence suggesting that intervention on both risk factors and education could contribute to health improvements. Extensions of such approaches to composite scores, SEP, behaviors, and health outcomes would be straightforward and would help identifying the direct and indirect effect of the scores on health.

## Economic Downturn: The Evidence From Lifepath

A primary objective of *Lifepath* was to assess the impact of changes in measures of socioeconomic position caused by the 2008 economic recession on health and biomarkers in Europe. The focus of this work was on Ireland and Portugal—examples of European countries strongly affected by the economic recession. We have first focused on the impact of changes in socioeconomic circumstances resulting from the recession on the health of children during the build-up phase of aging. Then we focused on evaluating impacts of the recession on the health of adults during the decline phase of aging. Taken together, our work contributes to understanding how changes in socioeconomic position during periods of economic decline affect the biology and health outcomes of children and adults in European countries.

### Key Findings

Detailed results that we only summarize here will be later submitted for publication.

**The Great Recession had sizeable impacts on families and children**, particularly in Ireland, where about two thirds of families experienced a significant or very significant effect of the recession in 2011-2013. The recession had the largest impact on disadvantaged families, disproportionally affecting parents who had lower income, education, and occupational grade. The most common forms of economic hardship families suffered as a result of the recession included reductions in wages, hours worked, and social welfare benefits; difficulties affording basics; and job loss. Comparing Irish and Portuguese cohorts, economic impacts of the recession on households appeared to be stronger in the former.

**The effects of the recession on physical health and biological markers appear to be strongest for children and adolescents**. In Ireland, changes in household socioeconomic circumstances increased the risk of poor child health, asthma, and rapid weight gain during infancy and childhood, while in Portugal, parental job loss was associated with worse cardiovascular biomarkers and anthropometric outcomes. Evidence from Finland also suggested that the recession increased the risk of mental health problems as measured by a rise in the use of psychotropic medication among children and adolescents.

The recession impacted physical health among adults as well, but negative effects were less consistent and weaker than those observed for children. Reductions in wages associated with the economic recession in Ireland led to increased overweight and obesity among mothers, but job loss and other major changes did not have any negative measurable health effects; in some cases, they may have led to some health improvements, such as reductions in smoking among mothers who lost their job. Likewise, we found no evidence of effects of the recession on adults from the EpiPorto cohort in Portugal. Among older Irish adults, cross-sectional analyses revealed that job loss prior to retirement was associated with a worse biomarker profile, and higher wealth was associated with a better clinical profile. However, longitudinal analyses revealed that changes in housing wealth associated with the recession were not associated with significant changes in older people's health.

Changes in access to affordable diets as a result of the recession appear to be critical mechanisms linking recession to child normal growth and health. While our cohorts did not have data on dietary habits, the most consistent effect of the Irish recession on children relates to difficulties with affording food and basic needs, which translated into increased risk of overweight and obesity. In Portugal, parental job loss during the recession was associated with increased LDL cholesterol, total cholesterol, triglycerides, fat mass, BMI, overweight, and waist to height ratio in children at age 7, all of which are outcomes potentially reflecting changes in the quality of diets. Likewise, in Ireland, wage reductions associated with the economic downturn increased the risk of overweight and obesity among mothers. These results highlight the importance of interventions to prevent the risk of rapid growth during infancy and development of obesity and metabolic syndrome in children and adults through social policies that support families in purchasing healthy foods, particularly focusing on vulnerable families.

**Economic downturns have consistent negative effects on the mental health of both adolescents and adults**. In Ireland, changes in economic circumstances in the household (pressure due to changes in employment, wages, working hours, welfare, and material deprivation) had a significant negative effect on child psychological adjustment. In Finland, worsening local economic conditions led to an increase in the use of antidepressant medication among adult women and anxiolytic-sedative-hypnotic medication among adult men. Among adolescents, father's unemployment led to a significant increase in psychotropic medication use. These findings corroborate earlier evidence and further highlight potential consequences for long-term mental health risks and health systems.

Although economic downturns negatively influence mental health, we found no evidence that these effects translate into immediate physical health or biological changes. A common hypothesis is that economic strain generates permanent alterations of stress activation of the autonomic nervous system and the hypothalamic-pituitary-adrenocortical axis. However, we found little evidence of an increased stress response for adults or children experiencing major changes in their own or their parents' employment, income, and consumption during the recession. In Ireland child psychological adjustment during the recession was primarily impacted directly by the recession and resulting household economic pressures, rather than indirectly through family stress. Overall, effects on health and biomarkers appear to be dominated by changes in material circumstances, leading, among other things, to worsening in diet-related outcomes such as overweight and obesity.

**There are significant gender differences in the impact of the economic recession on children**. Data from Portugal suggests that girls may be more likely to gain weight and develop overweight, obesity, and a higher waist to hip ratio as a result of parental job loss; while boys exposed to parental job loss during the recession experienced higher triglycerides levels. These differences may reflect differences in the metabolic response of boys and girls to changes in their immediate social environment during the recession.

### Conclusions

Overall, our findings suggest that changes in social and economic circumstances during the recession had negative implications for the health of children and adolescents. Negative effects on physical health and biomarkers appear to be more pronounced in those exposed to the recession in early life than in late adolescence or adulthood. By contrast, there is less consistent evidence that the economic recession impacted the physical health and biomarker profile of adults, including parents, working adults and older people in Ireland and Portugal. Our results, nevertheless, confirm that economic shocks induced by the economic recession are associated with worsening mental health and reduced psychological well-being during both the build-up and the decline phases of life. Whether mental health effects translate into changes in physical health, however, was less clear, as we found no evidence of consistent effects of economic shocks on stress-related biomarkers.

Overall, most of the impacts of the recession on physical health among children and adults appear to be confined to changes in their material circumstances and their impact on their ability to afford healthy foods influencing their risk of overweight, obesity and metabolic syndrome. Our results emphasize the need to protect vulnerable families and children against food insecurity during economic recessions through appropriate food supplementation or broader social protection programmes that may help prevent vulnerable families from adopting unhealthy diets during periods of economic hardship.

Further details will be provided in our papers in preparation.

## Health Impact Assessment Through Microsimulation Models

A growing literature is advocating for public health interventions and policies in order to reduce health inequalities and long-term incidence of chronic diseases (85, 87–91). Tools such as evaluation models can be particularly useful to inform and support policy makers in their decisions. Health care evaluation models have been defined as “analytic methodologies that account for events over time and across populations, that are based on data drawn from primary and/or secondary sources, and whose purpose is to estimate the effects of an intervention on valued health consequences and costs” (92). These tools give quantitative estimations which help prioritize decisions: several competing health policies can be defined and compared, for example policies aiming at different ages in life, or policies which could be combined in broad prevention plans or applied as single specific interventions.

A large body of evidence—including from *Lifepath*—emphasizes the importance of the early years of life in constructing health in adulthood. The predictive value of socio-economic environment during pregnancy as well as the socio-economic position (SEP) of the parents has been shown (93, 94). Early life socioeconomic circumstances were shown to contribute to social inequalities in adult mortality (95) and adult morbidity. In *Lifepath* analyses, links between early socio-economic environment and various health variables later in life have been reported: children's height trajectories differed according to maternal education (96), disadvantaged socioeconomic position (SEP) in childhood was associated with chronic kidney disease in adulthood by a critical period effect (26), and early life material deprivation at 9 months was associated with a higher risk of Epstein Barr virus among 3-year old children (25). At the biological level, childhood SEP was associated with a sustained upregulation of the inflammatory transcriptome (81), and maternal education was shown to have a potential influence on child's methylome at birth and adolescence (40). The association between maternal education or parental occupation and allostatic load measured during adulthood was mediated through education, material factors, and health behaviors (6).

In the continuity of the socioeconomic position in the family during childhood and adolescence, the individual socio-economic position of a young adult as measured by educational attainment is a predictor of both future socioeconomic position and future health behaviors (97). Associations between individual educational attainment and smoking later in life have been described (98). Lifepath analyses showed in multicohort studies how socioeconomic position was associated with mortality (7) or loss of physical functioning at older ages (8) independently of multiple risk factors such as smoking, physical inactivity, obesity, or alcohol intake. Health behaviors (mostly smoking) were also shown to contribute to socioeconomic inequalities in health (74).

The choice to intervene on fundamental or on proximal causes of diseases should be based on long-term results rather than on the evidence based on short-term studies. Along the same lines, assessing the priority level of interventions in early life compared to interventions in adulthood, based on experimental designs, would require long-term studies which are extremely difficult and costly. Therefore, quantification of long-term results using models and simulations might be a valuable approach to give different levels of priorities to interventions and policies targeting various age groups or various health determinants. Microsimulation models have been developed and used by suppliers of health insurance, health care services, and government to guide their choices (99).

These models have to face complexity. A single intervention is not realistic: several interventions would more probably be combined at different periods along the life-course. In addition, consistency between macro-, meso- and micro-levels of interventions is a prerequisite for achieving optimal effects. For example, local programs or interventions designed to reduce social inequalities in health would be useless in a situation where macro-policies lead to an increase in inequalities. Quantitative information on the expected benefits from different strategies is thus needed to fuel the debate and inform decisions on priorities. In particular, the quantification of expected results might provide information on the potential results of policies outside the institutional health sector compared to intervention within the health sector. Similarly, results of individual-level interventions vs. system-level interventions, or long-term effects of interventions for children vs. short-term results of interventions in adults (example: anti-smoking campaigns) should be assessed.

In *Lifepath* we have used microsimulation based on individual data from cohorts. The causal relationships have been described using directed acyclic graphs. From these models, it is thus possible to use a counterfactual approach in order to simulate an intervention. Using this approach might provide some different results from other approaches estimating the contribution of a given factor using multiple linear models (74). For example, tools like population attributable fractions (PAF), defined as “the proportional reduction in average disease risk over a specified time interval that would be achieved by eliminating the exposure of interest from the population while distributions of other risk factors in the population remain unchanged” (100), are considered to provide “useful information for health planning and setting health priorities by creating a hierarchy of risk factors” (100). However, PAF usually ignore the sequential ordering of risk factors. The classic calculation of PAF involves adjusting for “independent” factors, however if a factor is in fact a mediator of the association between an early exposure and the outcome, adjusting for this factor may be misleading: the causal effect of the early exposure may disappear after adjustment, leading to consider this exposure as non-relevant in terms of causality, whereas both factors are involved in the causal chain and may potentially constitute relevant targets for developing interventions [(101), submitted].

The results for our analyses suggested that:

- Promoting interventions in early life, such as improving the educational and childhood conditions (social conditions of the parents, ACE and member's educational attainment in results from the NCDS 58 cohort, and participant's educational attainment in results from GAZEL) might have positive consequences on the future health of children. This benefit might be of the same order of magnitude as interventions to reduce smoking in adulthood.
- The estimated effect of counterfactual interventions to improve social conditions or smoking seemed to be slightly stronger in men than in women.
- Estimations from the NCDS 58 cohort showed that counterfactual interventions on Adverse Childhood Experiences (ACEs), participant's educational level and smoking seemed to have stronger effects among people whose parents have low educational level (except for the intervention on smoking among women).

The methodology used in this work has some advantages. It allows simulating various interventions at different levels of effectiveness, assessing the impact on health and inequalities in health. These results have to be discussed in relation to the following considerations.

- The impact on health depends on the prevalence and the relative risk related to the exposure. Some exposures were frequent, such as parent's low social position (around 60% to 65%) or participant's low educational level (around 80%). Smoking had a lower prevalence, around 20 to 33%. In contrast, the frequency of ACEs (11%) was rather low in the NCDS 58 cohort. Consequently, counterfactual interventions decreasing by half the prevalence of exposure to parent's low social position or participant's low educational level were expected to have stronger effects than interventions decreasing by half the prevalence of ACEs.
- One limitation is that the effect of interventions was studied one by one or combining only two interventions, in contrast to the real world in which interventions can occur at different levels, different periods of time and eventually in combination in a complex system.

### Conclusion

Microsimulations may contribute to transfer knowledge from research to policy and help decision making, especially to compare the potential long-term effects of different policies. Information is needed about the potential benefits of interventions and policies in different age groups, or on the potential interest of combining several interventions. These methods could help to take into account complexity of the real world, including distal as well as proximal factors.

The method we used relied on real data from cohorts and could be implemented for more or less complex causal models, with a transparent communication of causal assumptions. Our preliminary results indicated that policies promoting interventions in early life on ACEs and education could have effects as large as the effects expected by interventions on smoking in adulthood. Some of the results also seemed to show the potential interest of proportionate universalism, but were not clearly replicated in both cohorts under study.

Estimates obtained from microsimulation models provide what could be expected from a given policy, defined in very general terms: to act on social characteristics in early life, or to act on behaviors such as smoking in adulthood. These models provide estimates to meet general policy objectives and to identify the best potential target for interventions, but say nothing about which kind of interventions should be implemented and how they could be implemented. Research is also needed to accurately describe interventions and policies so that their transfer to another context could be possible and effective.

## Policies to Address Health Inequalities, and the Example of ACEs

### Policies to Address Health Inequalities

Policies on health and social care tend to be divided into universal and targeted policies. A universal policy is one that applies to interventions that are available to the whole population. For example, in several European countries health care is available to all at the point of need, free of charge and regardless of income. Universal policies benefit everybody but at high cost to national budgets. Some argue that if people can afford to pay they should do. However, this could produce two tier systems where there are high quality private services for the more affluent and poor quality services for the less affluent.

Targeted policies often have the goal of reducing inequalities. So, for example, conditional cash transfers are targeted at vulnerable low-income families. A third approach is proportionate universalism which is where universal services are resourced and delivered at a scale and intensity proportionate to the degree of need. Services are therefore universally available, not only for the most disadvantaged, but they are provided proportionately to the level of presenting need.

The evidence from *Lifepath* is that interventions to reduce disadvantage are needed in childhood to support healthy aging through the life-course but current older generations also need health and social care interventions to support good health in old age. Table 4 summarizes possible universal and targeted policies that impact the life-course. This list is not exhaustive but is included to illustrate where epidemiology studies on health inequalities can inform health and social care policies.

**Table 4:** Universal and targeted health and social care policies for four age groups (Early Years 0–4, Childhood 5–18, Working age 19–66 and 67 years+; from www.lifepathproject.eu).

	**Early years 0–4**	**Childhood 5–18**	**Working age 19–66**	**67 years +**
Universal	Universal health care	Universal health care	Universal health care	Universal health care
	Child benefit (UK)	Child benefit (UK)		State pension
	Immunization	Immunization		Winter Fuel Payments (UK)
		Universal education		
			Health&Safety/Occupational Health	
	Smoke free public places	Smoke free public places	Smoke free public places	Smoke free public places
	Smoke free private cars with children in	Smoke free private cars with children in	Smoke free private cars with children in	Smoke free private cars with children in
	Sugar tax	Sugar tax	Taxes on alcohol, tobacco, and sugar	Taxes on tobacco, alcohol, and sugar
	Ban on hydrogenated trans fats	Ban on hydrogenated trans fats	Ban on hydrogenated trans fats	Ban on hydrogenated trans fats
	Food labeling—calories, traffic lights	Food labeling—calories, traffic lights	Food labeling—calories, traffic lights	Food labeling—calories, traffic lights
	Promote active transport (walking, cycling)	Promote active transport (walking, cycling)	Promote active transport (walking, cycling)	Promote active transport (walking, cycling)
	Promote safe play spaces for children (indoor and outdoor)	Promote safe play spaces for children (indoor and outdoor)		
	Replace private transport with high quality public transport (trains, buses, light railway, subway)	Replace private transport with high quality public transport (trains, buses, light railway, subway)	Replace private transport with high quality public transport (trains, buses, light railway, subway)	Replace private transport with high quality public transport (trains, buses, light railway, subway)
Targeted at low SEP	Female children and CKD (Ireland)—what was the cause?		Conditional cash transfers	Flexible working practices which make work more attractive than retirement
	Provision of suitable housing (space and free of damp and pollution)	Provision of suitable housing (space and free of damp and pollution)	Provision of suitable housing (space and free of damp and pollution)	Provision of suitable housing (space and free of damp and pollution)
			Working tax credit (UK)	
			Job seekers allowance (UK)	
			Employment and Support Allowance (UK)	
	Social care (CYP)	Social care (CYP)	Social care (adult)	Social care (elderly)
			Universal credit (UK)	
	Emergency support during recessions	Emergency support during recessions	Emergency support during recession	Emergency support during recession
			Cold weather payments	Cold weather payments
	Free travel for under 5 s		Travel payments for job seekers	Free bus travel

### Evidence for the Effectiveness of Policies in Addressing ACEs

The *Lifepath* Project had two core objectives: first, to understand the mechanisms through which SEP changes biological function with consequences for premature aging and mortality risk. Second, to provide updated, relevant, and innovative evidence for the development of future policy to reduce health inequalities. We have provided evidence for the effectiveness of policy interventions which seek to reduce exposure to adverse environments either directly, by reducing the differential risks associated with SEP, or indirectly, by altering the entry to disadvantaged SEP. There are a very large number of possible policy interventions and risk exposures which could be examined, but trying to do so would be impossible. Instead, based on analysis of the literature we focused on a key area of policy intervention: income and resource supplementation. Similarly, rather than looking at the effectiveness of income and resource supplementation on all risk exposures, we focus solely on adverse childhood experiences (ACEs).

There is now overwhelming evidence that exposures in childhood influence adult health outcomes (102, 103) and the mechanisms and pathways through which this influence works are becoming established (104). A related literature has been accumulating on the specific ACEs that are important and their likely consequences for later socioeconomic status (105, 106), health behaviors, and health outcomes (107, 108). The scientific literature has been steadily building up a detailed understanding of the determinants of adult disease and health inequalities and the manner in which these are structured across the life-course. We now understand more fully, not only the proximate causes of disease, but what Geoffrey Rose (109) termed the “causes of the causes,” that is, the “upstream” social environments experienced by individuals and families that give rise to a higher risk of disease acting through material exposures, unhealthy behaviors or psychosocial mechanisms. The policy literature has increasingly recognized the importance of social and economic environments early in life in the etiology of disease and in the formation of inequalities in health and mortality (110, 111) and has proposed a large number of policy options to reduce the burden of disease. This literature repeatedly underlines the role of social protection systems as a key policy tool in reducing health inequalities. Yet, as far as we know, there has been no systematic review to date which has examined the effectiveness of interventions on income and resources that may be available to reduce children's exposure to a broad set of adverse experiences in early life.

**Figure 12:**
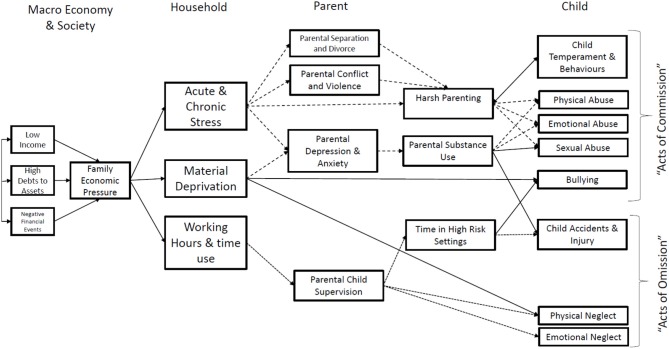
Distal and proximal influences on adverse childhood experiences.

Following Berger and Waldfogel (112), a priori, there are five main pathways through which low income in the micro-environment of the child, particularly the immediate family, can influence the probability of adverse experiences. These are presented schematically in Figure 12. Four domains are specified running left to right which are the nested ecological domains which structure the probability of adverse childhood experiences: macro-economy and society, household, parent, and child. Here, the line of causality runs from left to right—macro-economy to child experiences, but child characteristics also play a role and interact with the parent level.

We have conducted a review on the interventions aimed at reducing the impact of SEP on ACEs. For inclusion, studies had to include an intervention, defined as an intentional change in policy or practice, the objective of which is to increase the resources available to households and families. Such interventions will include (but are not restricted to):

- Income supplementation and maintenance (including in-work programs such as the Earned Income Tax Credit, tax rebates)
- Conditional Cash Transfers
- In-kind benefits (e.g., food stamps)
- Financial assistance for at-risk groups (e.g., ex-prisoners)
- Housing allowances (utilities and housing support)

Studies which utilize exogenous change in income and resources via “natural experiments” have not been included in the review. The review only included randomized or quasi-randomized studies that met the minimum methodological criteria defined in the Cochrane EPOC:

- Controlled “before and after” studies will have at least two or more sites in each intervention arm;
- Intervention and control groups are collected contemporaneously;
- Intervention and control sites are comparable;
- Interrupted time series will have at least three time points before and after the intervention and a clearly defined intervention point.

Based on previous research, we included cohort studies that have controlled for confounding (for example through matching), or reverse causation (for example, using instrumental variable analysis, regression discontinuity designs (RDD) or difference-in-differences). The ACEs we considered were Childhood physical abuse; Household substance abuse; Childhood sexual abuse; Household mental illness; Exposure to domestic violence; Emotional, psychological or verbal abuse; Parental separation or divorce; Household criminality; Neglect; Family financial problems; Family conflict or discord; Bullying; Death of parent or close relative or friend; Separation from family (e.g., out-of-home care); Serious childhood illness or injury.

Our systematic review included studies (*n* = 25) that used experimental or quasi-experimental methods to examine the relationship between the interventions which targeted family resources and ACEs (35). Among the studies identified, 48% showed a significant effect, of which 83% are a protective influence on the probability of specific ACEs. The results of this review point to the potential of upstream interventions based on resource allocation in reducing ACEs, but also to broad variations in impact, and considerable gaps in knowledge. More than one third of studies in our sample were randomized-controlled trials, i.e., the gold standard for the evaluation of the impact of this kind of programme on childhood circumstances. The rest of the studies were based on strong quasi-experimental designs to infer causal effects of the policies on ACEs. Differences in study designs also point to differences in the scope and ambition of these programmes.

Quasi-experiments are *a posteriori* evaluations, most often in one sample, of variations in existing wider policies (e.g., extending eligibility to the US EITC, Earned Income Tax Credit), more often than stand-alone programmes evaluated experimentally. As noted (113), these designs are robust but also limit translation to other national contexts. Our results also point to broad regional variations in terms of the type of programs considered and their effects on ACEs. All conditional and unconditional cash transfers were evaluated in middle-income countries, most notably in Latin-American countries. Most of the evidence on income related interventions has been produced in high-income countries and especially in the US. Only one study focused on a low-income country and future research should focus on filling the knowledge gap in less developed countries.

It is worth noting that these upstream interventions were not designed to prevent or reduce exposures to ACEs. However, most of the studies in our sample described an unintended protective effect of income-related interventions on childhood experiences. Our review contributes to the literature linking upstream economic interventions to a wide range of socio-economic and health outcomes (including for example educational attainment, employment, or child nutrition) (114–116). We argue that this line of work could be usefully extended to include childhood experiences.

Studies included in our review covered a range of ACEs, but few studies used comparable outcomes. Although we include ACEs based on previous literature, such diversity limits the comparability of the results across investigations. In addition, the majority of papers focused on a single ACEs, though previous research shows that influential ACE are more likely to be multiple. Nonetheless, it is likely that interventions which support family and household income will act on the common factors which contribute to ACE, and so reduce the risk of multiple as well as single ACEs (117). These limitations underscore the need for a better harmonization of ACE measures. This work is underway in the literature exploring the association between early childhood experiences and later life health (118) and could be usefully extended to the interventional literature.

Global estimates of the health care costs attributable to child maltreatment are not yet available, but Fang et al. (119) have estimated that in the East Asia and Pacific Region they amount to 2% of gross domestic product annually. If we were to add in the economic costs of educational failure, unemployment, work absence, and reduced earnings which have also been shown to be associated with adverse childhood experiences (105), it is likely that the full cost will be considerably higher. Our findings suggest that, even if we set aside for one moment the toll of adverse childhood experiences in terms of human suffering, the economic consequences alone mean that governments should be directing more resources at prevention and alleviation. The World Health Organization's global plan for the prevention and control of non-communicable diseases (NCDs) targets seven major risk factors (5) and focuses policy on health behaviors, regulation and pricing, yet an argument could be made from the results of this review, that there should also be a shift in our focus toward the early life experiences that may predispose some individuals and groups toward worse health behaviors as well as having a direct effect on the risk of NCDs via neglect and activation of glucocorticoid and adrenergic signaling pathways (120).

In conclusion, evidence presented in our review shows that upstream interventions have a role to play in preventing ACEs. As these policies are not targeted at ACEs, they should not be considered in isolation but complemented, for example, with psychosocial programmes to develop resilience. However, this review demonstrates the importance of upstream interventions targeting the early drivers of later life outcomes. Future efforts to build the evidence base on economic interventions to reduce ACEs should implement rigorous impact evaluations using experimental or quasi-experimental methods on a validated set of ACEs.

Concerning the tools to be used to tackle social disparities in health in early years, we refer e.g., to the work of Sarah and Mark Redding on the Pathways Community HUB model. This model uses Community Health Workers to address the socioeconomic inequalities in a community. Similar models have been developed in other countries (121, 122).

## Lessons from RCTs and Non-Experimental Intervention Studies

One of the key aims of *Lifepath* was to understand how changes in socioeconomic position (SEP) influence health and aging. We contributed to this aim by studying the health effects of two types of changes in SEP: changes in SEP generated by the financial crisis, and changes in SEP generated by social policy. Research summarized in this chapter examines the impact of a series of social policies on the health of socially disadvantaged adults and children, providing evidence on potential policies that might contribute to reducing health inequalities. The report examines impacts of both *targeted* and *sectoral (untargeted)* policies. *Targeted policies* explicitly aim to reduce poverty, for example, by providing conditional cash transfers and work tax credits to poor families and individuals with the explicit aim to reduce short-term or long-term poverty. By contrast, *sectoral policies* are untargeted and aim to achieve broader changes in the distribution of economic resources, for example, by increasing social mobility through compulsory schooling laws that require young people to stay longer in school as a way to improve their future life chances.

First, we report the results of analyses of the impact of one targeted antipoverty policy evaluated using a Randomized Controlled Trial (RCT) design, particularly a conditional cash transfer in the city of New York (NYC). Second, we focus on a selection of both targeted and sectoral policies evaluated using a quasi-experimental design applied using observational data from *Lifepath* and other cohorts. Taken together, these analyses offer important and at times surprising insights into how changes in socioeconomic position generated by a wide range of policies affect biology and health outcomes of children and adults.

### Key Findings

We summarize here still unpublished results and some published findings (41–44, 123).

**A randomized controlled trial design can offer critical insights into the short-term effects of changes in SEP on health**. Social policies are rarely designed with the explicit aim to influence health, and instead focus on impacts on social outcomes such as poverty, employment, and education. Using data from a randomized experiment, *Lifepath* is one of the first studies to show that it is possible to use randomized controlled trials of social policy to examine how changes in SEP, particularly income, influence the health of adults and children in the short- to medium-term.

**Conditional cash transfers in NYC improved overall health and mental well-being of adults by improving financial well-being**. Using data from the NYC Family Rewards experiment (118), we were able to document improvements in psychological well-being, as well as small but significant changes in self-rated health in response to this programme. Nevertheless, changes in self-rated health appeared to be short-lived and were not sustained a few years after the programme ended. By contrast, improvements in psychological well-being appear to take time to accrue and were only observed 42 months after the study ended.

Conditional cash transfers in NYC had small or no effects on the health insurance coverage and use of health services, but they had sizeable effects on the use of dental care, a service that is not typically covered by standard health insurance packages. Evidence from NYC Family Rewards suggests that conditional cash transfers might not offer sufficient incentives to change health care use behavior, partly because many participants already had health insurance and regular contact with a physician. It is in the realm of care that is not typically covered by health insurance, particularly dental care, that conditional cash transfers may be more effective, increasing use of preventive dental care by a margin that could have important implications for oral health later in life.

While evidence of effects of NYC conditional cash transfers on physical health is weak, there appear to be substantial heterogeneity and an indication that adults experiencing multidimensional disadvantage (in terms of socioeconomic, employment, and health status) at baseline did experience important positive changes in their physical health in response to the transfers. Evidence from the Family Rewards experiment suggests that the programme reduced Body Mass Index as well as the probability of reporting high-blood pressure and high cholesterol among disadvantaged respondents, some of which were sustained over several years. These results underscore the value of conditional cash transfers in improving the health of the most disadvantaged adults.

*Lifepath* analysis of two major educational policy reforms in France and the UK confirm that compulsory schooling laws can improve cognitive aging outcomes, but they raise concerns about the unintended negative consequences of increased compulsory schooling on mental health. Both the ROLSA and Berthoin reforms (43) increased years of schooling in post-war France and the UK, and evidence for France suggests that these changes translated in improved cognitive function later in life. However, there was no evidence of impact on measures of physical functioning, and among women, increased compulsory schooling may have led to increased depressive symptoms later in life. These findings highlight the need to carefully consider the potential limits of policies that solely increase the length of compulsory schooling as strategies to improve mental health.

A law that increased years of compulsory schooling in France did not translate into better biomarker profiles (44) in adulthood, questioning the potential of educational policy to change the biological course of disease. Using a wide array of biomarkers assessed in Constances, a large sample of adults in France, *Lifepath* analyses suggest that increased years of compulsory schooling had no effects on a wide range of biological markers, and it may have led to increased blood pressure and white cell counts, suggesting the prospect of worsening biological outcomes. Although results do not necessarily question the premise that education leads to better health, they cast doubt on the effectiveness of compulsory schooling laws as strategies to reduce the biological risk of disadvantaged groups. However, the results of these studies conducted in one country may not be generalizable in other school systems.

Analyses from *Lifepath* suggest that non-contributory/social pensions—which provide cash transfers to poor older adults without a contributory pension—may lead to small but significant improvements in overall health and hospitalization rates among men. Using data for Colombia, which implemented a major programme of cash transfers to older adults living below a poverty threshold, we were able to show some small improvements in health and reductions in hospitalizations. However, these effects were relatively small in magnitude and were confined to older men only, suggesting the need to identify ways to maximize the health benefits of cash transfers to poor older people in low-and-middle-income countries.

Among children in Colombia, conditional cash transfers increased contact with preventive services, improved dietary diversity scores and reduced risk of thinness, but they also increased body mass index (41, 42). Overall, the positive effect of conditional cash transfers appears to be more consistent in the context of a middle-income country such as Colombia, where the risk of poor nutrition and reduced access to care is still prevalent and higher than in the context of the United States, where effects of the programme were weaker. Yet, our findings also raise the need to monitor potential negative effects of cash transfers on body mass index, and they underscore the need to consider the prevention of non-communicable diseases in the design of conditional cash transfer programmes in low-and-middle-income countries.

**The well-documented benefits of home ownership for mental health extend to those who acquire a home later in life**. Using data from the US Health and Retirement Study and applying a fixed-effects approach, our analyses show that acquiring a home after age 50 is associated with a reduction in depressive symptoms. These findings indicate that policies that help older people access homeownership may bring mental health benefits in older age. These results also add to the growing recognition that home-ownership in older age may have public health implications for current and future generations of older Americans (unpublished).

### Conclusion

Overall, these findings offer a mixed picture of the potential of social policies to reduce health inequalities. On the one hand, findings from experimental studies suggest that conditional cash transfers and work tax credits may improve the psychological well-being of low-income adults, but they also suggest that effects on physical and overall health assessments of adults and children are weak or inconsistent in the short- to medium-term. While these results may be due to the relatively short time window of assessment in the RCTs evaluated, they may also suggest that policies that increase income for poor adults may not be sufficient to erase a lifetime of exposure to poverty, at least not immediately. It may also seem surprising that studies consistently find a strong relationship between income and health, yet changes in income generated by policy do not always lead to changes in health. These findings suggest that at least some of the relationships might be due to reverse causality (i.e., those who are sick are more likely to be poor due to the negative effects of illness on the ability to work and accumulate income) or unmeasured confounding. Therefore, from our analysis, it is not certain that conditional cash transfers and work tax credit policies can “prevent” or “remedy” the adverse health effects of poverty.

Findings from quasi-experimental studies suggest that social policies may sometimes be effective in changing the distribution of education or income, and through these changes they may improve cognitive aging and positively influence some physical health and functioning outcomes. Yet, our analyses also offer a cautionary tale of the dangers of changing the distribution of socioeconomic position without considering potential negative mental health effects on those affected. Our analyses consistently showed that although compulsory schooling laws increased the length of schooling and in some cases educational attainment, they may also have led to unexpected increases in depressive symptoms, and some negative effects on biological markers of diseases. These results question a simple causal interpretation of the relationship between socioeconomic position and health, and question the use of observational evidence as basis to propose specific policies to reduce inequalities for which we have limited empirical evidence on their health impact. Overall, our findings suggest that changes in socioeconomic position may not always lead to expected improvements in population health, and they emphasize the need to monitor how specific social policies influence the health and aging trajectories of individuals and families.

## General Conclusions: Biography and Biological Capital

There is still a wide gap between social and natural sciences, both on methodological and conceptual grounds. Natural sciences focus in particular on biological mechanisms and outcomes, i.e., they address “*zoe*” (biological life), while social sciences address “*bios*” (biographical life), if we refer to the terminology used by Ronald Dworkin. In fact, epidemiologists aim to connect *zoe* and *bios* in meaningful ways, though this attempt has rarely become explicit. An exception is the work of Nancy Krieger who proposed the concept of “embodiment.” *Biology and biography* (124) meet in the health status of an individual, depending on social position at a given age. These concepts start to be incorporated into epidemiological research, via the integration of social contexts and biomarkers in a life-course approach. The results from analyses carried out within *Lifepath* suggest that the socioeconomic environment, from early life and across the life-course, is an important risk factor for health and exerts its effects via intermediate biological mechanisms.

## Author's Note

Lifepath website: some of the material presented here can also be found in the Lifepath website www.lifepathproject.eu.

## Author Contributions

All authors listed have made a substantial, direct and intellectual contribution to the work, and approved it for publication.

## The LIFEPATH Consortium (in alphabetical order)

Harri Alenius, Mauricio Avendano, Henrique Barros, Murielle Bochud, Cristian Carmeli, Luca Carra, Raphaele Castagne, Marc Chadeau-Hyam, Francoise Clavel-Chapelon, Giuseppe Costa, Emilie Courtin, Michaela Dijmarescu, Cyrille Delpierre, Angelo D'Errico, Pierre-Antoine Dugue, Paul Elliott, Silvia Fraga, Valerie Gares, Graham Giles, Marcel Goldberg, Dario Greco, Allison Hodge, Michelle Kelly-Irving, Piia Karisola, Mika Kivimaki, Vittorio Krogh, Thierry Lang, Richard Layte, Benoit Lepage, Frances Macguire, Johan Mackenbach, Michael Marmot, Cathal McCrory, Roger L. Milne, Peter Muennig, Wilma Nusselder, Salvatore Panico, Dusan Petrovic, Silvia Polidoro, Martin Preisig, Olli Raitakari, Ana Isabel Ribeiro, Fulvio Ricceri, Oliver Robinson, Jose Rubio Valverde, Carlotta Sacerdote, Vincenzo Salerno, Roberto Satolli, Gianluca Severi, Terrence Simmons, Silvia Stringhini, Rosario Tumino, Anne-Clare Vergnaud, Paolo Vineis, Peter Vollenweider, Marie Zins.

## Conflict of Interest

The authors declare that the research was conducted in the absence of any commercial or financial relationships that could be construed as a potential conflict of interest.
